# Ghost spintronic THz-emitter-array microscope

**DOI:** 10.1038/s41377-020-0338-4

**Published:** 2020-06-08

**Authors:** Si-Chao Chen, Zheng Feng, Jiang Li, Wei Tan, Liang-Hui Du, Jianwang Cai, Yuncan Ma, Kang He, Haifeng Ding, Zhao-Hui Zhai, Ze-Ren Li, Cheng-Wei Qiu, Xi-Cheng Zhang, Li-Guo Zhu

**Affiliations:** 1 Institute of Fluid Physics, China Academy of Engineering Physics, Mianyang, 621900 Sichuan China; 2 Department of Optics and Optical Engineering, University of Science and Technology of China, Hefei, 230026 Anhui China; 3 Microsystem & Terahertz Research Center, China Academy of Engineering Physics, Chengdu, 610200 Sichuan China; 4 Beijing National Laboratory for Condensed Matter Physics, Institute of Physics, Chinese Academy of Sciences, 100190 Beijing, China; 5 National Laboratory of Solid-State Microstructures and Department of Physics, Nanjing University, Nanjing, 210093 Jiangsu China; 6 Department of Electrical and Computer Engineering, National University of Singapore, 4 Engineering Drive 3, Singapore, 117583 Singapore; 7 The Institute of Optics, University of Rochester, Rochester, NY 14627 USA

**Keywords:** Super-resolution microscopy, Terahertz optics, Super-resolution microscopy, Terahertz optics, Super-resolution microscopy

## Abstract

Terahertz (THz) waves show great potential in nondestructive testing, biodetection and cancer imaging. Despite recent progress in THz wave near-field probes/apertures enabling raster scanning of an object’s surface, an efficient, nonscanning, noninvasive, deep subdiffraction imaging technique remains challenging. Here, we demonstrate THz near-field microscopy using a reconfigurable spintronic THz emitter array (STEA) based on the computational ghost imaging principle. By illuminating an object with the reconfigurable STEA followed by computing the correlation, we can reconstruct an image of the object with deep subdiffraction resolution. By applying an external magnetic field, in-line polarization rotation of the THz wave is realized, making the fused image contrast polarization-free. Time-of-flight (TOF) measurements of coherent THz pulses further enable objects at different distances or depths to be resolved. The demonstrated ghost spintronic THz-emitter-array microscope (GHOSTEAM) is a radically novel imaging tool for THz near-field imaging, opening paradigm-shifting opportunities for nonintrusive label-free bioimaging in a broadband frequency range from 0.1 to 30 THz (namely, 3.3–1000 cm^−1^).

## Microscopy: imaging ‘ghosts’ more clearly with terahertz waves

A modification of the technology called terahertz near-field microscopy brings improvements in resolution and speed for biological and medical imaging and nondestructive materials testing. Terahertz waves lie between the microwave and infra-red regions of the electromagnetic spectrum. The improved ‘ghost-imaging’ procedure was developed by researchers in China, Singapore and the USA, led by Li-Guo Zhu at the China Academy of Engineering Physics. In ghost imaging the illuminating radiation is split into one beam that interacts with the object being studied and another beam that does not. The so-called ghost image of the object is then constructed by computational comparison of the different behaviour of the two beams. The innovation depends on gaining enhanced control of the structure of the terahertz radiation using a system called a spintronic terahertz emitter array.

## Introduction

The unique properties of terahertz waves (0.1–10 THz)^1,2^, such as the nonionizing photon energy, spectral fingerprint, and transparency for most nonpolar materials, have attracted much research interest and enabled many applications, such as nondestructive testing^3^, biodetection^4–6^, and cancer imaging^7^. However, the long wavelength of THz waves (1 THz ~ 300 μm) typically limits the resultant imaging resolution to greater than the millimetre scale in conventional far-field imaging methods due to the well-known Rayleigh diffraction limit, therefore restricting their use in many emergent applications such as cellular imaging. On the other hand, near-field imaging by mapping the object-modulated evanescent waves paves the way towards deep subwavelength resolution and is particularly desirable at long wavelengths, for example, at THz frequencies. AFM- or STM-tip-enhanced THz probes^8,9^ or microantenna/aperture THz probes^10^ have achieved micrometre- or even atomic-scale resolution^9^. Nevertheless, these techniques require mechanical raster scanning of the surface of an object pixel by pixel, with a relatively poor signal-to-noise ratio (SNR). Recently, near-field ghost imaging techniques^11–14^ have been experimentally demonstrated to increase the SNR by $\sqrt N$ times (where *N* denotes the pixel number of a digital picture) over that in raster scanning^15^. In these approaches, THz images are spatially encoded in the near field with deterministic patterns (e.g., the Walsh-Hadamard matrix^11–17^). Then, the total intensities (or electric field amplitudes) of encoded THz images are collected and detected with a single-pixel detector in the far field. After postprocessing via computational algorithms to correlate the detected THz intensities (or electric field amplitudes) with the deterministic patterns^18,19^, near-field images can be reconstructed. In this scheme, the subwavelength spatial information “hidden” in the diffracted far-field distribution can be recovered from the intensities (or amplitude fields) recorded by a mere single-pixel detector.

To encode THz images in a single pixel, the conventional method is to use photogenerated spatial THz wave modulators as reconfigurable masks^11–13^. However, the THz wave amplitude passing through the subwavelength apertures of modulators follows the scaling rule 1/*a*^3^ (where *a* denotes the diameter of the apertures on the mask)^20^, which fails to image deep subwavelength structures.

Alternatively, directly detecting THz images^14^ or generating patterned THz waves^16,17^ by encoding femtosecond (fs) laser pulses in nonlinear electrooptic (EO) crystals (such as ZnTe) has been proposed to bypass the scaling rule. However, subwavelength structures are rapidly blurred upon propagation in hundreds of micrometre-thick EO crystals^14^ (Supplementary Fig. S1). Recently, the time-resolved single-pixel detection of THz pulses has been theoretically proposed^16^ and experimentally demonstrated^17^ to recover higher-resolution images. However, suffering from the milimeter-scale-thick nonlinear crystal, THz-wave generation along with diffraction occurs across the entire volume of the crystal, leading to a limited spatial resolution of 50–100 μm. Although the spatial resolution can be further improved by the inverse propagation algorithm, this requires full-wave measurements of THz pulses in the time domain with extra cost in terms of detection resources and involves an inverse problem. Furthermore, it only applies to recovering 2D-structured images. In addition, constrained by the electromagnetic boundary conditions (Supplementary Fig. S2), the distribution of the polarized THz field in subwavelength structures presents a “distorted” image^11–14^ that might lead to misrecognition of the object’s morphology.

In this work, we utilize a spintronic THz emitter (STE) to illuminate an object in the near field. STEs are a novel type of THz emitter based on the spin-related effects^21,22^ in ferromagnetic/nonmagnetic (FM/NM) heterostructures^23–26^, which are only a few nanometres thick but offer generation efficiency comparable to conventional milimetre-thick EO crystals. In principle, an STE can fully cover the 0.1–30 THz frequency range^24^ without phonon absorption, which is superior to all the current solid emitters. Limited by the 1-mm-thick ZnTe used as the detector (3-THz detection bandwidth) and the pulse duration of the pump laser (~90 fs), the STE in our work provided a bandwidth of up to 2 THz. To date, all applications of STEs have only focused on their far-field properties, whereas the highly efficient few-nanometre-thick STE is capable of illuminating an object at an extremely near field, which naturally breaks the diffraction limit. The key challenge for using STEs in near-field imaging is how to map the object-modulated THz field without near-field scanning probes. In view of this, we developed a near-field illuminating “array” (STEA) whose “elements” are coherent and individually programmable in binary states of either “on” or “off” by photoexcitation. Combining the programmable near-field illuminating STEA and far-field single-pixel detection, we designed and demonstrated a novel ghost spintronic THz-emitter-array microscope (GHOSTEAM) for THz wave imaging with deep subdiffraction resolution. A minimum resolution of 6.5 μm at a single pixel was demonstrated with a contrast of more than 57 ± 21% (>20% required by the Rayleigh criterion^14^) in a 6-µm metal gap. In addition, polarization effects on the subdiffraction-limited image were eliminated via postprocessing of two images with mutually orthogonal polarizations. In addition, TOF microscopic topography was demonstrated with a 3D silica structure.

## Results

### Concept design

**Fig. 1:**
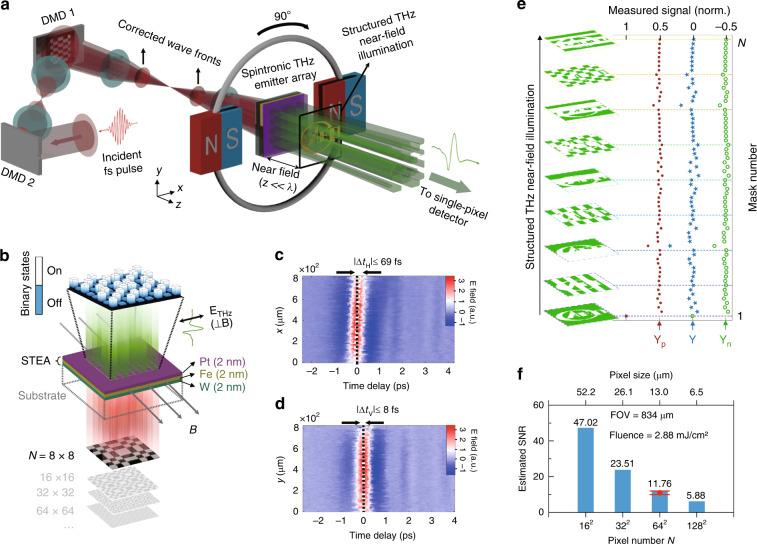
Schematic of the GHOSTEAM system. **a** Schematic of the GHOSTEAM system. The spintronic THz emitter array (STEA) is excited by two-DMD-encoded *fs* laser pulses and generates spatially coded THz pulses. An object “CAEP” was placed in the near-field region (z ≪ λ). The illuminating THz pulse was collected and sent to a single-pixel detector. **b** Schematic of the STEA, consisting of a W(2 nm)/Fe(2 nm)/Pt(2 nm) trilayer heterostructure and working in the binary emission state with polarization perpendicular to applied magnetic field ***B***. Spatiotemporal THz waveform along the horizontal (**c**) and vertical (**d**) directions. The wavefront is indicated by white dotted lines, and time *t* = 0 is indicated by black dotted lines. |Δ*t*_H_| and |Δ*t*_v_| are the temporal delays in the horizontal and vertical directions, respectively. **e** Schematic of the detection for ghost imaging, and measured signal **Y** (which is the difference between the positive mask value and negative mask value, **Y** = |**Y**_p_| − |**Y**_n_|, in the case of Hadamard multiplexing) from the single-pixel detector for an object “CAEP” illuminated by a sequence of prearranged structured THz waves. **f** Estimated SNRs as a function of pixel number *N* under the condition of a pump fluence of 2.88 mJ cm^−2^, FOV_1_ = 834 μm × 834 μm. The red marker with a value of 10.92 ± 0.97 is the experimental result (see the main text below and Supplementary section 4 for details).

In our design, as shown in Fig. 1a, the STEA under external magnetic field ***B*** generates spatially structured THz pulses upon fs-laser-pulse spatial photoexcitation. The STEA, consisting of a W(2 nm)/Fe(2 nm)/Pt(2 nm) trilayer heterostructure on a transparent MgO substrate (Fig. 1b), provides great THz conversion efficiency in terms of output amplitude, comparable to that of 1-mm ZnTe^24^. The STEA is capped with a 150-nm SiO_2_ layer (*n* = 1.97) to protect it from being damaged by the fs laser. The output THz electric field ***E***(*t*) is linearly polarized perpendicular to the applied magnetic field ***B***, as described by ***E***(*t*) ∝ (***J***_*c*_ = ***J***_*s*_ × ***B***)^21–26^, where ***J***_*s*_ represents the spin current induced by the fs laser and ***J***_c_ represents the charge current converted in the NM metals under ***B***. To perform ghost imaging (see “Materials and methods” for details), the Walsh–Hadamard matrix^27^ was used to code the STEA due to its unrivalled noise suppression performance among various measurement matrices^11–17^. The patterns, programmed in the order of the Walsh–Hadamard masks, were spatially encoded on the excitation fs laser beam by a digital micromirror device (DMD) with a switching time of 5 μs. On the exit surface of the STEA, the profile of the output THz pulse is as accurate as that of the excitation fs laser because the 150 nm propagation distance in the SiO_2_ protective layer is too thin for the THz wave to be diffracted (150 nm ≈ 5 × 10^−4^λ_0_/*n*, where λ_0_ = 600 μm and *n* = 1.97; see Supplementary Fig. S1 for the theoretical calculation of the near-field evanescent wave). The spatial near-field profile of the THz pulse from the STEA consists of individually real-time programmable “elements” up to 128 × 128 (see Supplementary Fig. S3) in either “on” or “off” binary emission states, as shown in Fig. 1b. The pixel size of each “element” is 6.5 μm × 6.5 μm in our experiments, which can be zoomed in or out by using an optical projection imaging system depending on the practical requirements. After being transmitted through an object placed in the near-field region (*z* = 150 nm), the structured THz pulses were collected and focused onto a 1-mm-thick (110) ZnTe crystal for single-pixel coherent detection by EO sampling. The peak electric amplitudes were recorded for reconstructing THz subdiffraction ghost images.

After reflection upon a DMD, the fs laser pulse suffers from wavefront tilt in the DMD deflection direction (horizontal) since the DMD is essentially a reflective blazed grating^28^. The resultant fs laser pulse with a tilted wavefront will badly lose its temporal coherence among individual micromirrors along the deflection direction. To correct the wavefront tilt, another DMD (DMD_2_) was imaged onto the encoding DMD (DMD_1_) by a 4*f* system with a 1:1 imaging ratio, as shown in Fig. 1a. Due to the fixed time delay of ghost imaging (corresponding to the peak of the THz pulses), the DMD-induced temporal smearing should be minimized. Otherwise, decoherence (large time delay in the wavefront) would result in severely distorted images (see Supplementary Fig. S6). The temporal delay in the horizontal direction |Δ*t*_H_| was ultimately corrected to less than 69 ± 13 fs (Fig. 1c) but could not be completely eliminated due to the manufacturing tolerance of the tilt angles of the two DMDs (see Supplementary Fig. S6). Meanwhile, the temporal delay in the vertical direction |Δ*t*_V_| was measured to be less than 8 fs (Fig. 1d), which is expected because the two DMDs only deflect in the horizontal direction. The spatiotemporal THz waveforms (Fig. 1c, d) were measured by ghost imaging using 64-order Walsh–Hadamard coding 1-D masks (see “Materials and methods” and Supplementary Fig. S5 for detailed information and Supplementary GIFs. 1 and 2 for raw data).

An object was illuminated by the coherent programmable STEA using a sequence of prearranged Walsh–Hadamard masks, and the computational ghost imaging algorithm was applied to correlate the peak amplitude measured by a single-pixel detector with the sequence of predetermined incident THz wave patterns (Fig. 1e, with the detailed procedure shown later). The reconfigurable area on the STEA, defined as the first imaging field of view FOV_1_, was measured as 834 μm × 834 μm, with up to 128 × 128 coding pixels. The SNRs of reconstructed images encoded by Walsh–Hadamard masks were numerically determined as a function of the pixel number (or pixel size in FOV_1_; see “Materials and methods”), which gave a reasonable value of ~47.02 when FOV_1_ was coded into 16 × 16 pixels under our experimental conditions (pump fluence ~2.88 mJ cm^−2^, dynamic range of peak field ~1043, and pulse fluctuation ~0.7%), as shown in Fig. 1f. It is worth noting that although the detected THz band was less than 2 THz due to the 90-fs pulse and 3-THz detection bandwidth (1-mm ZnTe), the STE has demonstrated complete coverage of the entire THz region, i.e., 0.1–30 THz, when a shorter pump pulse (10 fs) and a wide-band detector are used^24,26^. More experimental details are given in “Materials and methods” and Supplementary section 2.

### Subdiffraction ghost imaging

**Fig. 2:**
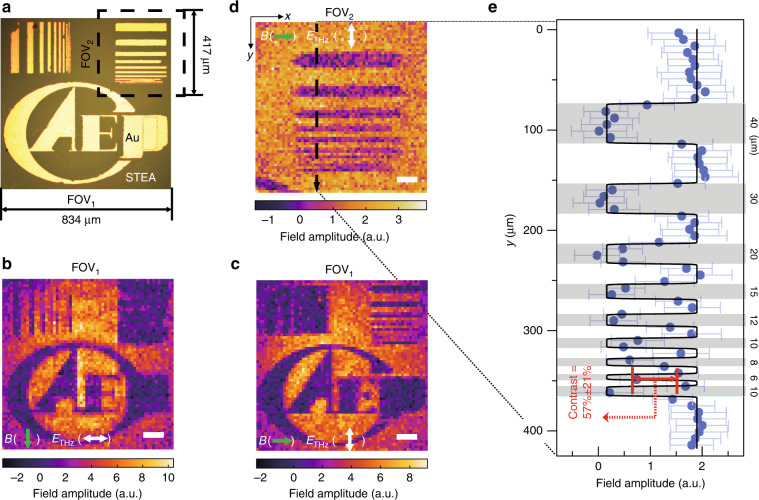
Subdiffraction-limited images from GHOSTEAM. **a** Optical image of an object with a field of view of FOV_1_ = 834 μm × 834 μm. The bright regions are gold attached on the 150-nm-thick protective SiO_2_ layer on top of the STEA. THz ghost images in FOV_1_ with a magnetic field (green arrows) applied along the vertical direction (**b**) and horizontal direction (**c**). The pixel size and scale bar are 13.0 μm and 100 μm, respectively, for both images. **d** THz ghost image in FOV_2_ (indicated by the black dashed box in a) with a pixel size of 6.5 μm and a scale bar of 50 μm. The applied magnetic field ***B*** is along the horizontal direction (indicated by the green arrow), and the polarization of the THz radiation (indicated by the white double-headed arrow) is perpendicular to ***B***. **e** Averaged amplitude of the THz field along the black dashed arrow in (**b**). Blue dots are averaged experimental data, and the black solid curve is the fit to the Boltzmann sigmoidal function (see Supplementary section 5 for details). Grey areas represent the metal regions, with corresponding widths indicated. A contrast ratio of 57 ± 21% is observed at the 6-μm width metal slit.

The STEA was experimentally reconfigured in a 64 × 64-order Walsh–Hadamard matrix sequence to acquire THz subdiffraction-limited images of an object (see Fig. 2a) positioned *z* = 150 nm from the STEA (thickness of the protective SiO_2_ layer on top of the STEA). The field amplitudes of spatially coded THz waves that passed through the object were measured at a fixed time delay of 0 ps (as indicated by the black dotted lines in Fig. 1c, d). The reconstructed 64 × 64 ghost images in FOV_1_ = 834 μm × 834 μm with mutually orthogonal polarizations are shown in Fig. 2b, c, whose pixel sizes are both 13.0 μm × 13.0 μm. The reconstructed 64 × 64 ghost image in a smaller area FOV_2_ = 417 μm × 417 μm (indicated by the black dashed box in Fig. 2a) with a smaller pixel size of 6.5 μm × 6.5 μm is shown in Fig. 2d. The *y*-dependent THz field distribution across the slit region (indicated by the black dashed arrow in Fig. 2b) was extracted from the associated pixels, as shown in Fig. 2e. Note that every pixel value was averaged from the identical-row pixels within the slit region, with prior knowledge about the object’s horizontal homogeneity in the slit region. A contrast ratio of 57 ± 21% (>20% required by the Rayleigh criterion^14^ for distinguishing slits) was observed in the narrowest 6-μm metal slit within FOV_2_ (see Supplementary section 5 for the quantification process and Supplementary GIFs. 3–5 for the raw data), which clearly proves that our GHOSTEAM system deeply breaks the diffraction barrier of ~366 μm for the 600-μm THz wave (Rayleigh resolution of 0.61λ_0_/NA). The spatial resolution of the GHOSTEAM system using this experimental setup is limited to the minimum available pixel size of 6.5 μm, which depends on the accuracy of the mask patterns on the STEA projected by the DMD. As the micromirrors of the DMD are arranged in the diamond orientation, the accuracy of the coding profile on the STEA is decreased since a mask pixel is formed by fewer micromirrors (see Supplementary Fig. S3). Regardless of the diffraction of the coding optical light, the resolution of GHOSTEAM is limited by the propagation distance between the emission surface of the STEA and the object (the thickness of the protective 150-nm-thick SiO_2_ layer in this experiment), which is theoretically estimated as submicrometer (see Supplementary Fig. S1).

### Polarization-free image

**Fig. 3:**
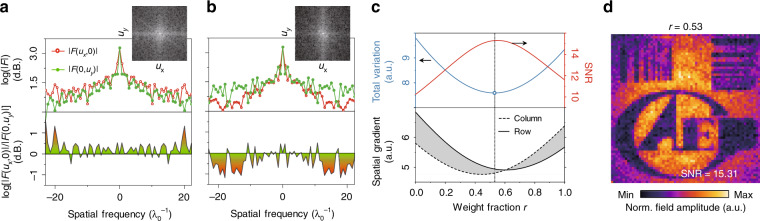
Polarization-free THz image. 2D Fourier transform of Fig. 2b (**a**) and 2c (**b**). The amplitude |*F*(*u*_*x*_, *u*_*y*_)| (|*F*(*u*_*x*_, 0)| and |*F*(0, *u*_*y*_)| are in red circles and blue dots, respectively) is shown as a function of the spatial frequency (upper panels). log[|*F*(*u*_*x*_, *0*)|/|*F*(*0*, *u*_*y*_)|] is shown in the lower panels for comparison of the horizontal and vertical spatial distributions. **c** Assessments (total variation and spatial gradient; see Supplementary section 7 for details) of the fused image as a function of weight fraction *r*. The blue open circle indicates the minimum TV = 7.59 obtained at *r* = 0.53. **d** Fused polarization-free THz image with optimized SNR = 15.31 at *r* = 0.53.

Polarization impacts the subwavelength imaging (see the dark region where the slits are along the wave polarization in Fig. 2b, c), which is expected due to the electrical field boundary conditions at the subwavelength scale^11,13,14^ (see Supplementary Fig. S2) and hinders accurate resolution of the object’s morphology. With advancement of the in-line rotating THz wave polarization by an external magnetic field, we secured two images with mutually orthogonal polarizations. This feature allows us to achieve a higher-contrast polarization-free image through postfusion of the two images. To do this, 2D Fourier transform was applied to Fig. 2b, c individually to quantify the distribution in the spatial frequency domain |*F*(*u*_*x*_*,u*_*y*_)| along the *x* and *y* directions. The amplitude |*F*(*u*_*x*_*,u*_*y*_)| as functions of spatial frequencies *u*_*x*_ and *u*_*y*_ is shown in Fig. 3a, b, which indicates loss of the high spatial frequency (>10/λ) components parallel to ***E***_THz_ in the polarization images. To acquire polarization-free THz image **X**_F_, the THz ghost images with horizontal polarization **X**_H_ (Fig. 2b) and vertical polarization **X**_V_ (Fig. 2c) were fused by the weighted average method, namely, **X**_F_ = *r***X**_H_ + (1 − *r*)**X**_V_, where *r* represents the weight fraction and lies within [0, 1]. The total variation (TV), which is a common assessment parameter for image clearness, was chosen to guide the optimization of the fusion process. The TV of **X**_F_ was calculated by

1 $${\mathrm{TV}} = \left( {\mathop {\sum }\limits_{i = 2}^{64} \mathop {\sum}\limits_{j = 2}^{64} {\left[ {\left( {\nabla _{\mathrm{H}}{\mathbf{X}}_{\mathrm{F}}\left( {i,j} \right)} \right)^2 + \left( {\nabla _{\mathrm{V}}{\mathbf{X}}_{\mathrm{F}}\left( {i,j} \right)} \right)^2} \right]} } \right)^{1/2}$$

where ∇_H_ and ∇_V_ are the discretized gradient operators along the horizontal and vertical directions, respectively. At the ratio of *r* = 0.53 in Fig. 3c, the minimal TV was achieved, and the corresponding optimal fused image is shown in Fig. 3d. In addition, the SNR (see Supplementary Fig. S13) and directional spatial gradients^29^ of the fused image are also given in Fig. 3c. The directional spatial gradients of **X**_F_ were used to quantify the spatial information of the fused image and were calculated by

2 $$G_{{\mathrm{row}}} = \left( {\mathop {\sum }\limits_i^{64} \mathop {\sum}\limits_{j = 2}^{64} {\left[ {\left( {\nabla _{\mathrm{H}}{\mathbf{X}}_{\mathrm{F}}(i,j)} \right)^2} \right]} } \right)^{1/2}$$

3 $$G_{{\mathrm{col}}} = \left( {\mathop {\sum }\limits_{i = 2}^{64} \mathop {\sum}\limits_j^{64} {\left[ {\left( {\nabla _{\mathrm{V}}{\mathbf{X}}_{\mathrm{F}}(i,j)} \right)^2} \right]} } \right)^{1/2}$$

As shown in Fig. 3c, the fused image at *r* = 0.53 shows more homogeneous spatial information in the vertical and horizontal directions, as visually presented in Fig. 3d, which proves that the polarization impacts are effectively overcome and that the original object’s morphology is better reproduced.

### Time-of-fight topography

**Fig. 4:**
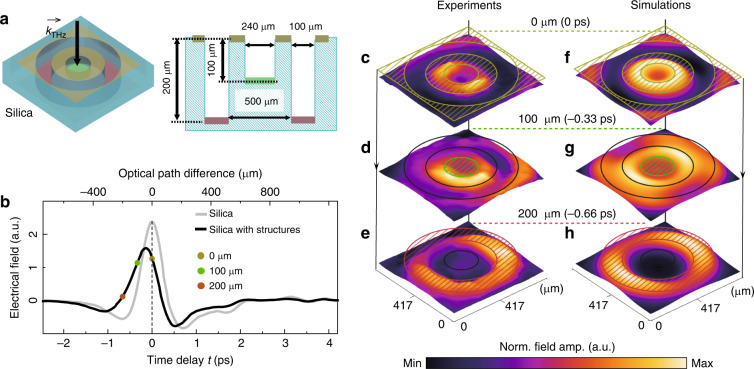
Time-of-flight THz microscopic topography using GHOSTEAM. **a** Structure of a prototype 3D object. The object with air grooves on SiO_2_ has three interfaces [indicated in yellow (upper), green (middle) and red (bottom)]. The arrow indicates the incidence direction of THz pulses with an illuminating area of FOV_1_. **b** Measured THz waveforms that passed through the sample within and outside the structured region. The solid dots indicate the EO sampling delay times of the TOF measurements with the GHOSTEAM system, corresponding to the three interfaces. Experimental subdiffraction-limited images in terms of height for the interfaces at 0 μm (**c**), 100 μm (**d**), and 200 μm (**e**). Note that experimental images were denoised using stationary wavelet transform (see “Materials and methods” for details). **f**–**h**, Simulated electrical distributions for the three interfaces relevant to **c**–**e**, respectively. The colour map for each image (**c**–**h**) is normalized individually.

Since the THz emission from the STEA used in our GHOSTEAM system is coherent, TOF measurements^30^ of THz pulses from the STEA can be adopted to enable microscopic topography of objects with depth resolution. TOF topography utilizes the optical path differences among different media to acquire the depth (*z* axis) distribution of a 3D object. For the sake of illustration, a 3D object made of silica by fs laser manufacturing (see “Materials and methods”) with three SiO_2_/air interfaces with air depths of 0, 100 and 200 μm (illustrated in Fig. 4a) was used to demonstrate TOF microscopic topography using the GHOSTEAM system. The structured silica was attached to the external surface of the STEA. Transient THz waveforms from the STEA that passed through the object within and outside the region of the trilayer structure were measured and are shown in Fig. 4b. Three THz ghost images relevant to the three interfaces, under the field of view FOV_1_, were measured at fixed time delays of 0, −0.33 and −0.66 ps (indicated by the three solid circles in Fig. 4b), as shown in Fig. 4c–e. The reconstructed images agree with the simulated electrical field distributions (Fig. 4f–h; see “Materials and methods” for simulation details). The subwavelength structures (scale of ~λ_0_/6) of the three interfaces are well resolved both experimentally and theoretically (note that diffraction leads to distortion in reconstruction of the middle interface).

## Discussion

In conclusion, we have presented a novel GHOSTEAM system for THz wave near-field microscopic topography. Compared with other existing THz wave near-field imaging systems, the GHOSTEAM system utilizes a real-time, reconfigurable, coherent STEA for structured near-field illumination. It was numerically and experimentally demonstrated that such a system can feature both micrometre-scale-resolved microscopy (≤6.5 μm) and depth-resolved topography. Further optimized optical projection imaging systems could enable submicrometre-scale resolution. Polarization-free subdiffraction-limited imaging was also achieved owing to the flexible tunability of the STEA.

The capacity of an STE to provide efficient THz pulses would further enable label-free cellular imaging or ultraprecise topography, a feature lacking in the current THz near-field imaging systems. STEs fabricated on flexible substrates^31^ would extend our GHOSTEAM system to imaging of objects with curved surfaces. Oscillator *fs* lasers have been demonstrated to drive STEs with a dynamic range above 60 dB (1000:1)^26^. Taking advantage of the MHz repetition rate and stable pulse energy of oscillators, the GHOSTEAM system is endowed with a much faster acquisition speed (in principle, an 18-fold improvement can be expected; see “Materials and methods” and Supplementary Fig. S9 for the estimation) than all other existing THz wave subdiffraction ghost imaging systems^11–14,16,17^, which require an amplified fs laser with a few-kilohertz repetition rate, leading to a slow acquisition speed and a complex system. In addition, using a shorter fs pulse and a wide-band detector, the ultrabroadband property of STEs, fully covering 0.1–30 THz frequencies^24^, has great potential for broadband THz applications. In addition, the GHOSTEAM system is compatible with compressive sensing (see Supplementary GIFs. 1–5), adaptive sampling^12,32^, parallel acquisition in *k*-space^33^ and coherent time-resolved full-wave detection in the time domain^16,17^.

## Materials and methods

### Experimental details

THz pulses (0.2–1.7 THz with a central frequency of 0.5 THz) were generated in W(2 nm)/Fe(2 nm)/Pt(2 nm) trilayer heterostructures [placed in a rotatable dc field (|***B***| = 80 mT)] by an 800-nm laser (duration of 90 fs, repetition rate of 1 kHz, and pump pulse energy of 20 μJ). The THz signals were electrooptically sampled by 1-mm-thick ZnTe (110) in combination with a balanced detector and were eventually recorded by a lock-in amplifier with an integration time of 100 ms (see Supplementary Fig. S4 for details). All the THz ghost images were multiplexed using the Walsh–Hadamard matrix and operated at respective fixed time delays (e.g., the peak time delays for Fig. 2b–d and appropriate time delays for Fig. 4c–e indicated in Fig. 4b). The acquisition time for each mask was 2 s, and the imaging time for each 64 × 64 ghost image (Fig. 2b-d) was ~4.5 h.

To optimize the wavefront of fs pulses, three steps were adopted to determine the positions of DMD_1_ and DMD_2_ (Wintech DMD4500, which contains 1140 × 912 diamond-arrayed micromirrors with a tilt angle of 12 ± 1° and a micromirror pitch of 7.6 μm). First, we acquired the one-to-one image of DMD_2_ by placing a CCD (see Supplementary Fig. S4c) at the presupposed position of DMD_1_ to adjust and determine the positions of DMD_2_ and two lenses (focal lengths of 100 mm). Second, by adjusting the position of DMD_1_, it could be determined when the images (at the presupposed position of the STE) shaped by DMD_1_ and DMD_2_ individually were simultaneously sharp. Third, their positions were accurately adjusted by measuring the waveforms of the Walsh–Hadamard masks of #2p and #2n (see Supplementary section 3) until reaching the minimal peak time delay difference between the two waveforms. The tilt angle difference between DMD_1_ and DMD_2_ was measured as 0.40° by comparing the zero-order diffraction angles of the two DMDs in the case of normal incidence.

### Computational ghost imaging

Let **O** represent the pixelated imaging target, consisting of *N* unknown elements **O**(*i*) at pixel *i*. **O** is a vector reshaped from the initial *L* × *L* image matrix **O**_m_, where *L* × *L* = *N*. The DMD is used to display the Walsh–Hadamard masks **ϕ**_1_, **ϕ**_2_, …, **ϕ**_*N*_ in sequence. **ϕ**_*i*_ with mask number *i* (1 ≤ *i* ≤ *N*) is an *L* × *L* matrix reshaped from the *i*th row of the *N*-order Walsh–Hadamard matrix **Φ**. Then, the correlation between the mask and object, which is recorded by a single-pixel detector, can be mathematically described by their inner product

4 $$y_i = < {\mathbf{\varphi }} _i,{\mathbf{O}} >$$

where **φ**_*i*_ is an *N*-length vector reshaped from **ϕ**_*i*_. The complete measurement vector is then given by

5 $${\mathbf{Y}} = {\mathbf{\Phi }} {\mathbf{O}}$$

In the experiments, the Walsh-Hadamard matrix consisting of “+1” and “−1” elements was realized by

6 $${\mathbf{\Phi}} = {\mathbf{\Phi}} ^{\left( {{p}} \right)} - {\mathbf{\Phi}} ^{\left( {{n}} \right)}$$

since the DMD can only modulate the amplitude of the incident light. In Equation (6), **Φ**^(p)^ is constructed by substituting all “−1” elements in **Φ** with “0”. **Φ**^(n)^ is acquired by **Φ**^(p)^ − **Φ**.

Ultimately, the ghost image can be reconstructed by

7 $${\mathbf{X}} = {\mathbf{\Phi}} ^{ - 1}{\mathbf{Y}} = {\mathbf{\Phi }} ^{ - 1}\left( {{\mathbf{\Phi}} {\mathbf{O}}} \right) = {\mathbf{O}}$$

### Spatiotemporal THz waveform mapping

Let ***E***(*ξ*, *t*) represent the THz spatiotemporal waveform to be measured, where *ξ* and *t* represent the spatial and temporal coordinates, respectively. ***E***(*ξ*, *t*) consists of *N* time-dependent vectors

8 $${\boldsymbol{E}}\left( {\xi ,t} \right) = \left| {\begin{array}{*{20}{c}} {{\boldsymbol{E}}_1\left( t \right)} \\ {{\boldsymbol{E}}_2\left( t \right)} \\ \vdots \\ {{\boldsymbol{E}}_N\left( t \right)} \end{array}} \right|$$

The sequentially recorded signals ***S***(*ξ*,*t*) can be written as

9 $${\boldsymbol{S}}\left( {\xi ,t} \right) = \left| {\begin{array}{*{20}{c}} {{\boldsymbol{s}}_1\left( t \right)} \\ {{\boldsymbol{s}}_2\left( t \right)} \\ \vdots \\ {{\boldsymbol{s}}_N\left( t \right)} \end{array}} \right| = {\mathbf{\Phi }}{\boldsymbol{E}}\left( {\xi ,t} \right)$$

Once the complete measurements are obtained, ***E***(*ξ*, *t*) can be calculated as

10 $${\boldsymbol{E}}\left( {\xi ,t} \right) = {\mathbf{\Phi }}^{ - 1}{\boldsymbol{S}}\left( {\xi ,t} \right)$$

In the experiments, the *i*th mask had *N* = 64 identical rows (columns) for spatiotemporal waveform mapping in the vertical (horizontal) direction, and every row (column) of the mask was the *i*th row of **Φ**. The spatial and temporal resolutions were 13 μm and 33 fs, respectively, for both Fig. 1c, d. The wavefront was then regarded as a linear fit of the peak time delay in ***E***_*i*_(*t*) to the corresponding spatial coordinate *ξ* (see Supplementary Fig. S5).

### Estimation of the SNR and potential frame rate of ghost images

The SNRs of ghost images obtained via Hadamard multiplexing (Fig. 1f) were estimated by the following equation (see Supplementary section 4 for the detailed mathematical derivation):

11 $${\mathrm{SNR}}_{\mathrm{H}} = \sqrt {\frac{{\it{k}}}{{2{\boldsymbol{N}}\left[ {{\it{\upgamma }}_{\mathbf{d}}^2 + \left( {{\it{\upgamma }}_{\mathbf{s}}/2} \right)^2} \right]}}}$$

where *γ*_d_ denotes the ratio of the dark noise to the THz peak, *γ*_s_ denotes the ratio of the peak root mean square error to the THz peak, *N* denotes the pixel number of the ghost image and *k* denotes the number of measurements for each mask. In our experimental setup, *γ*_d_ was measured as 1 × 10^−3^, and *γ*_s_ was measured as ~7 × 10^−3^, as indicated in Fig. 1f. Each mask value was averaged for *k* = 15. With these parameters, the SNR_H_ was estimated as 11.76 in the case of *N* = 64 × 64, according to Eq. (11).

The frame rate equals FPS = 1/(2*Nr*_c_*t*_mask_), where *t*_mask_ represents the acquisition time for each mask and *r*_c_ represents the compressive ratio. *t*_mask_ can be expressed as

12 $$t_{{\mathrm{mask}}} = 2N\left( {\gamma _{{\mathrm{d}}0}^2 + \gamma _{{\mathrm{s}}0}^2/4} \right)t_0{\mathrm{SNR}}_{\mathrm{H}}^2$$

where *t*_0_ denotes the pulse period and *γ*_d0_ and *γ*_s0_ denote the ratio of the dark noise to a single THz peak and the pulse fluctuation ratio within the “integration time” of *t*_0_, respectively. For an 80-MHz-oscillator-driven GHOSTEAM system, reasonable values are *t*_0_ = 12.5 ns, *γ*_d0_ = 2.83 (corresponding to a dark-noise-to-peak ratio of *γ*_d_ = 1 × 10^−3^)^26^, and *γ*_s0_ = 3.8 × 10^−3^ (see Supplementary Fig. S8). With these parameters and SNR_H_ = 11.76, the acquisition time 2*Nt*_mask_ for a 64 × 64 ghost image was calculated as ~15 min, yielding an imaging speed improvement by a factor of ~18 (4.5 h/15 min).

### Fs laser manufacturing

The silica sample with three air/silica interfaces for topography was manufactured by the femtosecond laser ablation method. In this method, a Ti-sapphire laser beam (800-nm central wavelength, 30-fs pulse width, 1-kHz repetition rate and 100-mW average power) was focused onto a silica substrate (1-mm thickness) using an objective lens (10×, numerical aperture NA = 0.25). The silica substrate travelled along multicircular trajectories at a speed of 100 μm/s (original radius *r*_0_ was 10 μm, interval between two adjacent circular trajectories Δ*r* was 10 μm, travel number *n*_r_ was 10, and the radius of the inner circular was *r* = *r*_0_ + Δ*r*(*n*_r_ − 1) = 100 μm). The laser ablation depth under the above parameters was ~50 μm, and the same ablation process was repeated to obtain an ablation depth of 100 μm, while the silica substrate was moved 50 μm up from the laser spot. Using the same laser fabrication method, the outer circular was produced with *R*_0_ = 250 μm, Δ*R* = 10 μm, *n*_r_ = 15, and *R* = *R*_0_ + Δ*R*(*n*_r_ − 1) = 400 μm. The process was repeated to obtain a deeper interface.

### Denoising and simulating near-field ghost topography

The results of near-field ghost topography (Fig. 4c–e) were denoised using the MATLAB toolbox Stationary Wavelet Transform Denoising 2-D. A five-level Haar wavelet was used to decompose the images. The selected threshold method was penalized low soft thresholding.

The electromagnetic field distributions as the THz pulse propagates in the near field (Fig. 4f–h) were simulated using the Wave Optics module of the commercial software COMSOL Multiphysics. Certain THz waves acquired in the experiment were set as the incident source, and the time-dependent solver was used to resolve the electromagnetic field distributions at discrete times (the time step was 33 fs). The study domain was a rectangle that was divided into silica (*n*_silica_ = 1.97) and air (*n*_air_ = 1), as illustrated in Fig. 4a.

## Supplementary information

Supplementary information for Ghost Spintronic THz-emitter-array Microscope

Supplementary-GIF1

Supplementary-GIF2

Supplementary-GIF3

Supplementary-GIF4

Supplementary-GIF5

## References

[1] Ferguson B, Zhang XC (2002). Materials for terahertz science and technology. Nat. Mater..

[2] Tonouchi M (2007). Cutting-edge terahertz technology. Nat. Photonics.

[3] Karpowicz N (2005). Compact continuous-wave subterahertz system for inspection applications. Appl. Phys. Lett..

[4] Yang X (2016). Biomedical applications of terahertz spectroscopy and imaging. Trends Biotechnol..

[5] Zou Y (2018). Label-free monitoring of cell death induced by oxidative stress in living human cells using terahertz ATR spectroscopy. Biomed. Opt. Express.

[6] Zou Y (2017). Terahertz spectroscopic diagnosis of myelin deficit brain in mice and rhesus monkey with chemometric techniques. Sci. Rep..

[7] Meng K (2014). Terahertz pulsed spectroscopy of paraffin-embedded brain glioma. J. Biomed. Opt..

[8] Huber AJ (2008). Terahertz near-field nanoscopy of mobile carriers in single semiconductor nanodevices. Nano Lett..

[9] Cocker TL (2013). An ultrafast terahertz scanning tunnelling microscope. Nat. Photonics.

[10] Kawano Y, Ishibashi K (2008). An on-chip near-field terahertz probe and detector. Nat. Photonics.

[11] Stantchev RI (2016). Noninvasive, near-field terahertz imaging of hidden objects using a single-pixel detector. Sci. Adv..

[12] Stantchev RI (2017). Compressed sensing with near-field THz radiation. Optica.

[13] Chen SC (2019). Terahertz wave near-field compressive imaging with a spatial resolution of over λ/100. Opt. Lett..

[14] Zhao JP (2019). Spatial sampling of terahertz fields with sub-wavelength accuracy via probe-beam encoding. Light. Sci. Appl..

[15] Watts CM (2014). Terahertz compressive imaging with metamaterial spatial light modulators. Nat. Photonics.

[16] Olivieri L (2018). Time-resolved nonlinear ghost imaging. ACS Photonics.

[17] Olivieri L (2020). Hyperspectral terahertz microscopy via nonlinear ghost imaging. Optica.

[18] Erkmen BI, Shapiro JH (2010). Ghost imaging: from quantum to classical to computational. Adv. Opt. Photonics.

[19] Edgar MP, Gibson GM, Padgett MJ (2019). Principles and prospects for single-pixel imaging. Nat. Photonics.

[20] Bethe HA (1944). Theory of diffraction by small holes. Phys. Rev..

[21] Valenzuela SO, Tinkham M (2006). Direct electronic measurement of the spin Hall effect. Nature.

[22] Sinova J (2015). Spin Hall effects. Rev. Mod. Phys..

[23] Kampfrath T (2013). Terahertz spin current pulses controlled by magnetic heterostructures. Nat. Nanotechnol..

[24] Seifert T (2016). Efficient metallic spintronic emitters of ultrabroadband terahertz radiation. Nat. Photonics.

[25] Feng Z (2018). Highly efficient spintronic terahertz emitter enabled by metal-dielectric photonic crystal. Adv. Optical Mater..

[26] Torosyan G (2018). Optimized spintronic terahertz emitters based on epitaxial grown Fe/Pt layer structures. Sci. Rep..

[27] Harwit M, Sloane NJA (1979). Hadamard Transform Optics..

[28] Murate K (2018). Adaptive spatiotemporal optical pulse front tilt using a digital micromirror device and its terahertz application. Opt. Lett..

[29] Eskicioglu AM, Fisher PS (1995). Image quality measures and their performance. IEEE Trans. Commun..

[30] Zhong H (2005). Nondestructive defect identification with terahertz time-of-flight tomography. IEEE Sens. J..

[31] Wu Y (2017). High-performance THz emitters based on ferromagnetic/nonmagnetic heterostructures. Adv. Mater..

[32] Phillips DB (2017). Adaptive foveated single-pixel imaging with dynamic supersampling. Sci. Adv..

[33] Chun IY, Adcock B (2017). Compressed sensing and parallel acquisition. IEEE Trans. Inf. Theory.

