# Supplementary material for: Ghost spintronic THz-emitter-array microscope
Source: Light Sci Appl. 2020 Jun 8;9:99. doi: 10.1038/s41377-020-0338-4 (PMC7280226; doi:10.1038/s41377-020-0338-4)
Supplement: Supplementary file 1 — Supplementary information for Ghost Spintronic THz-emitter-array Microscope [file 41377_2020_338_MOESM1_ESM.pdf]

Supplementary information for **Ghost Spintronic THz-emitter-array Microscope**

Si-Chao Chen[1,2,†], Zheng Feng[3,†], Jiang Li[1,3,†], Wei Tan[3,†], Liang-Hui Du[1,3], Jianwang Cai[4], Yuncan Ma[1], Kang He[5], Haifeng Ding[5], Zhao-Hui Zhai[1,3], Ze-Ren Li[1], Cheng-Wei Qiu[6], Xi-Cheng Zhang[7], and Li-Guo Zhu[1,3,*]

[1]Institute of Fluid Physics, China Academy of Engineering Physics, Mianyang, Sichuan 621900, China,

[2]Department of Optics and Optical Engineering, University of Science and Technology of China, Hefei, Anhui 230026, China,

[3]Microsystem & Terahertz Research Center, China Academy of Engineering Physics, Chengdu, Sichuan 610200, China,

[4]National Laboratory of Solid-State Microstructures and Department of Physics, Nanjing University, Nanjing, Jiangsu 210093, China

[5]Beijing National Laboratory for Condensed Matter Physics, Institute of Physics, Chinese Academy of Sciences, Beijing, Beijing 100190, China

[6]Department of Electrical and Computer Engineering, National University of Singapore, 4 Engineering Drive 3, Singapore 117583, Singapore

[7]The Institute of Optics, University of Rochester, Rochester, NY 14627, USA

†These authors contributed equally to this work.

*email: zhuliguo@tsinghua.org.cn

Section 1. Relevant factors of near-field imaging

Three factors impact the results of near-field ghost imaging, namely sensing distance which determines the spatial resolution, polarization which is relevant to the spatial information and the geometry of DMD which is relevant to the accuracy of coding profile on STEA.

Sensing distance

As sensing distance increasing from near field ($\ll \lambda$) to far field, the evanescent waves decay exponentially which results in the larger spatial resolution. In our case, i.e., wavelength λ_0 = 600 μm, refractive index n = 1.97 (SiO_2), and propagation distance z = 150 nm (thickness of the SiO_2 protective layer of our spintronic THz emitter), the critical spatial resolution is estimated as ~188 nm, as described in details below.

We assume that electromagnetic wave with wavelength of λ_0 transmits through two slits whose widths are both w and central positions are w apart from each other. The incident electric field is homogeneous with amplitude of 1. Then the field distribution is

$$E_0(x) = \mathrm{rect}[(x - w/2)/w] + \mathrm{rect}[(x + w/2)/w], \quad (1.1)$$

where $\mathrm{rect}[(x - a)/b]$ is the rectangle function with central position at a and width of b. The angular spectrum of $E_0(x)$ can be acquired by Fourier transformation

$$A_0(f_x) = \mathrm{F}[E_0(x)] = \int E_0(x) \exp(-\mathrm{i}2\pi f_x x)\, \mathrm{d}x, \quad (1.2)$$

where f_x represents the spatial frequency. According to the Helmholtz equation, the angular spectrum at z is given by

$$A_z(f_x) = A_0(f_x) \exp\left(\mathrm{i}kz\sqrt{1 - u_x^2}\right), \quad (1.3)$$

Where $k = 2\pi n/\lambda_0 = 2\pi/\lambda$ and $u_x \equiv f_x/\lambda$. The electric field at z can be acquired by inverse Fourier transformation

$$E_z(x) = \mathrm{F}^{-1}[A_z(f_x)] = \int A_z(f_x) \exp(\mathrm{i}2\pi f_x x)\, \mathrm{d}f_x. \quad (1.4)$$

If the two slits are well resolved, one can find a peak (with peak value of E_{peak}) in either slit and a valley (with valley value of E_{valley}) between the two slits. The contrast of $|E_z(x)|$ is defined as

$$\text{contrast} \equiv (E_{\text{peak}} - E_{\text{valley}})/E_{\text{peak}}. \quad (1.5)$$

To quantify the spatial resolution at given propagation distance z (150 nm), the contrast values at slit width varied from 2 nm to 1 μm were calculated using equations (1.1)—(1.5), as shown in Fig. S1a. According to the Rayleigh (Sparrow) criterion, two slits are barely resolved with a contrast equals to 20% (0%), and the corresponding critical slit width was calculated as w_R = 188 nm (w_S = 100 nm). The critical electric field amplitude $|E_z(x)|$ under Rayleigh (Sparrow) criterion is shown in Fig. S1b. Corresponding results with z = 10 nm (blue dashed curves) and z = 1 μm (orange dashed curves) are also given in Figs. S1a and S1b for comparison.

But note that the spatial resolution in our system is ultimately limited by the imaging accuracy of fs-laser (800 nm) on STEA, which is estimated at the scale of micrometers due to the diffraction limit of the 800-nm laser.

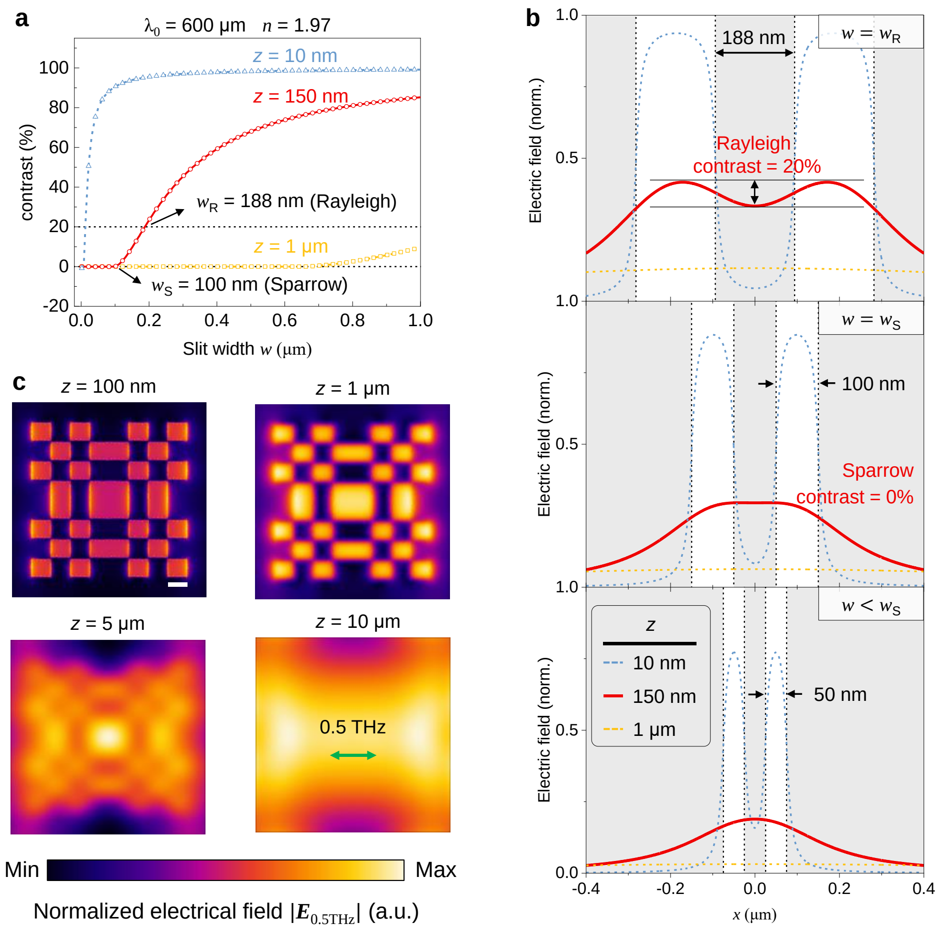

Figure S1| Impact of the sensing distance on spatial resolution. a, Calculated w-dependent contrast values at propagation distance of z = 150 nm (indicated in red). The wavelength is 600 μm and the refractive index of propagation medium is 1.97 (SiO_2). The critical contrast values required by Rayleigh criterion and Sparrow criterion are 20% and 0%, respectively, as indicated by two horizontal dashed lines. And the corresponding critical slit widths are w_R = 188 nm and w_S = 100 nm. **b**, The normalized electric field $E_z(x)$ transmitted through slit pairs with slit width of w_R (upper panel), w_S (middle panel), and 0.5w_S (lower panel) at propagation distance of z = 150 nm (red curves). Corresponding results with z = 10 nm (blue dashed curves) and z = 1 μm (orange dashed curves) are also given in (a) and (b) for comparison. **c**, Calculated diffracted profiles of a linearly polarized 0.5 THz field with a certain mask pattern at propagation distances of 100 nm, 1 μm, 5 μm and 10 μm. The polarization is indicated by the green double-headed arrow and the scale bar denotes 5 μm.

Polarization

Polarization has a significant impact on subwavelength imaging. As the feature (slit) size is longer than the wavelength, the transmittance (normalized by slit area A_{slit}) $T(A_{in}/A_{slit})$ of both p-polarized and s-polarized waves approach 1 with decreasing oscillation amplitude, as plotted in Fig. S2a. In Fig. S2b. We calculated the $T(A_{in}/A_{slit})$ for polychromatic p-polarized and s-polarized waves (0.1-1.5 THz) within slit width smaller than 600 μm. In subwavelength region (slit width < 60 μm), $T(A_{in}/A_{slit})$ of p-polarized wave is increasing sharply while $T(A_{in}/A_{slit})$ of s-polarized wave is decaying dramatically, which are consistent

with the experimental results (e.g., the region between letters “A” and “E” in Figs. 2b and 2c). Note that the averaged $T(A_{in}/A_{slit})$ of two polarizations approaches 1 at most feature size, that is the reason that fused image (Fig. 3d) is more homogeneous.

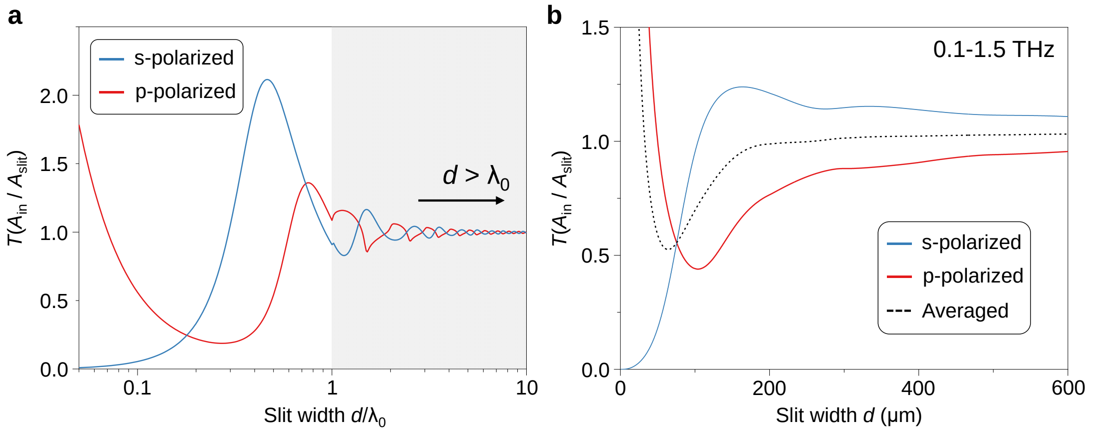

Figure S2| Impact of the polarization on Transmittance through metal slit. a, Simulated transmittance (normalized by slit area A_{slit}) $T(A_{in}/A_{slit})$ of monochromatic s-polarized and p-polarized waves as functions of slit width (normalized by wavelength). **b**, $T(A_{in}/A_{slit})$ of polychromatic s-polarized and p-polarized waves (0.1-1.5 THz) as functions of slit width within subwavelength scale. The black dashed curve is an average of the curves with mutually orthogonal polarizations.

Impact of diamond micromirror array on coding

Due to the diamond-arrayed micromirrors of the DMD[1], the accuracy of coding profile on STEA would decrease when a mask pixel is formed with less micromirrors, as illustrated in Fig. S3. It is a fatal factor to our ghost imaging, notably in the case of projected pixel size smaller than 6.5 μm (pixel number corresponds to 128 × 128 in FOV_1), as shown in Fig. S3d.

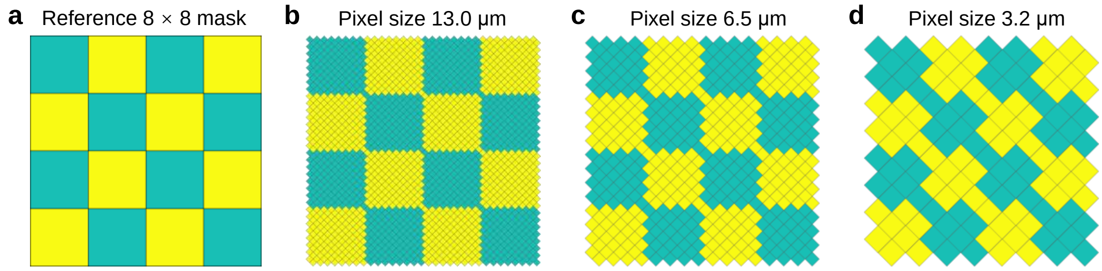

Figure S3| Impact of diamond micromirror array on coding accuracy. a, A reference mask with 8 × 8 pixels. **b-d**, Schematics of the 8 × 8 mask displayed on DMD with projected pixel size of 13.0 μm (**b**), 6.5 μm (**c**) and 3.2 μm (**d**). Each micromirror is approximately 7.6 μm in size and arranged in diamond orientation.

Section 2. Experimental details

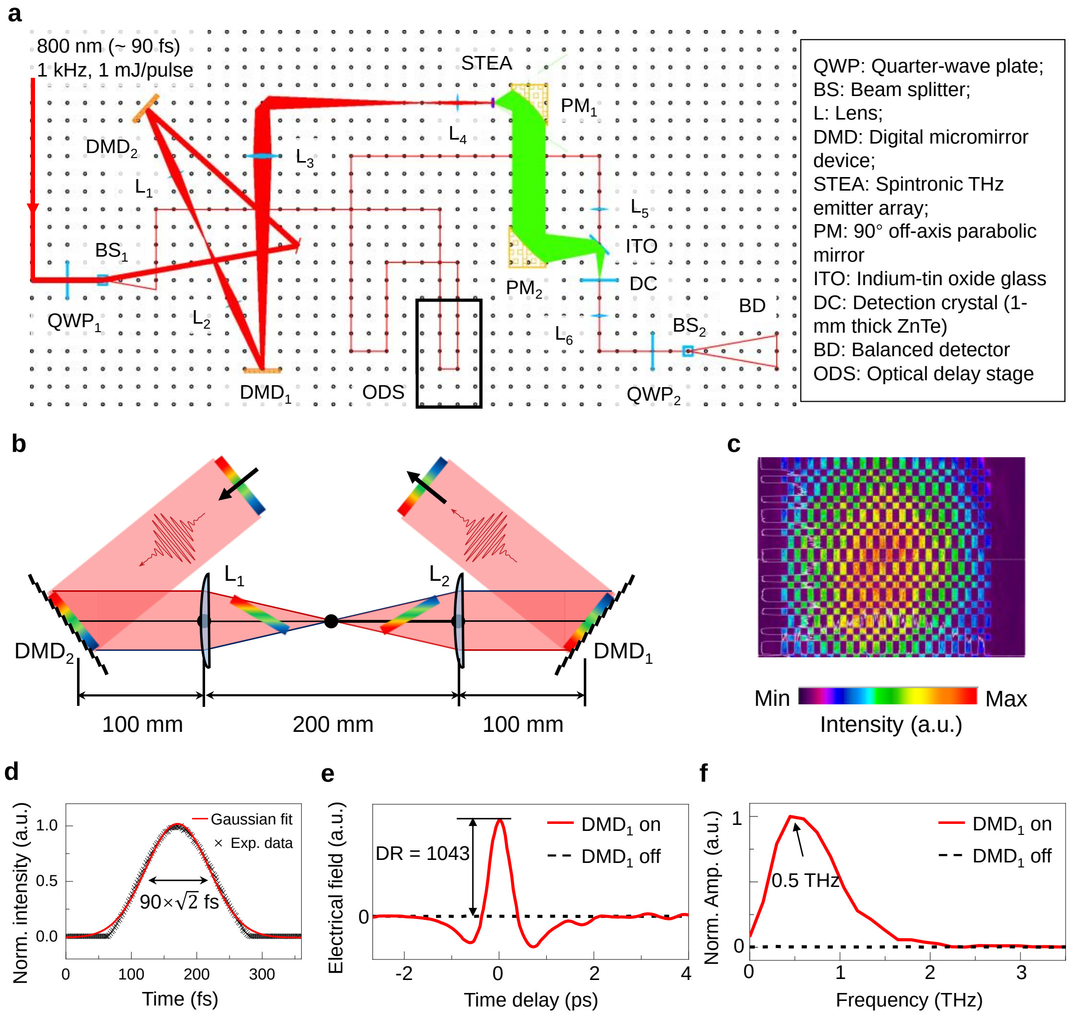

Figure S4| Experimental details. a, Schematic of GHOSTEAM. **b**, Wave front correction. Wave front is tilted with angle of ~12° in every reflection by on-state DMD and two lenses (focal length of 100 mm) were used to correct the wave front, as described in the main text. The wave fronts are colored in Rainbow to distinguish their directions. **c**, One-to-one Image of DMD_2 (with some certain mask displayed) at the position of DMD_1 acquired by CCD. **d**, Femtosecond pulse recorded by autocorrelator. The pulse duration was measured as about 90 fs. **e-f**, Transient THz waveforms (**e**) recorded by lock-in amplifier (LIA) and the corresponding spectrum (**f**). The red solid lines and black dashed lines indicate that the DMD_1 states are all on and all off, respectively. The measurements were operated with pump power of 20 mW (DMD_1 on), pump area of 834 μm × 834 μm, repetition rate of 1 kHz and LIA's integration time of 100 ms. The dynamic range (DR) was measured as 1043 and the central frequency was 0.5 THz.

Section 3. Wave-front measuring and impact on fixed-time ghost imaging

It is critical to make the wave front of pump pulse as flat as possible to cancel out the temporal smearing added by DMDs, since the ghost imaging was operated at some certain fixed time delay.

Wave-front measuring

One can see Supplementary GIFs. 1 and 2 for full raw data and the spatiotemporal THz waveform maps reconstructed with sub-sampled data.

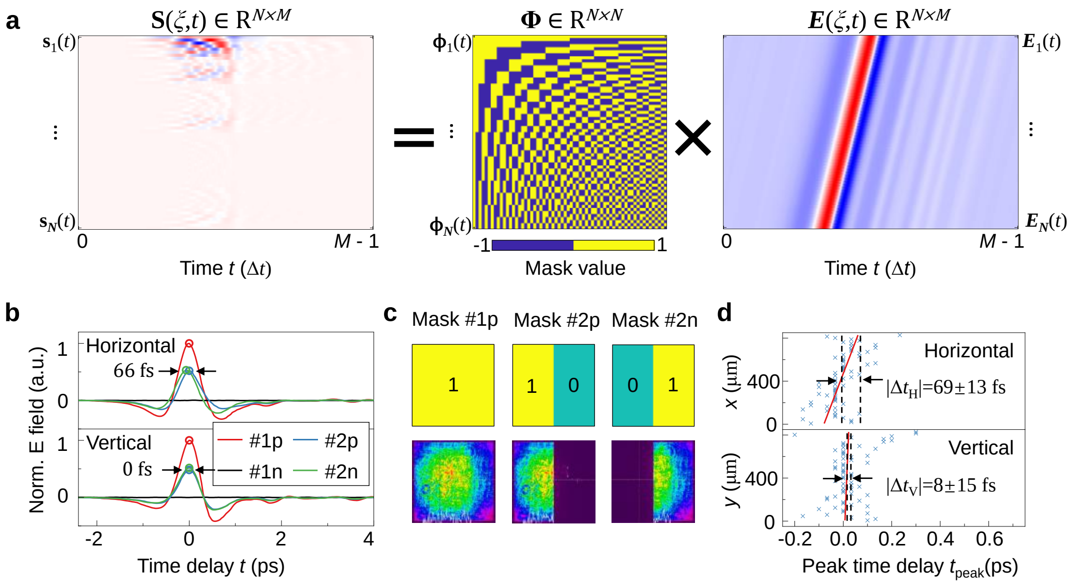

Figure S5| Wave-front measuring. a, Algorithm for spatiotemporal THz waveform mapping. The measured signal matrix $\boldsymbol{S}(\xi,t)$, measurement matrix $\boldsymbol{\Phi}$ (Walsh-Hadamard matrix) and electrical waveform matrix $\boldsymbol{E}(\xi,t)$ are illustrated in the left, middle and right panels, respectively. Both $\boldsymbol{S}(\xi,t)$ and $\boldsymbol{E}(\xi,t)$ consist of N rows and M columns, where each row and column represent a temporal and a spatial vector, respectively. **b**, Normalized experimental waveforms excited with masks #1p, #1n, #2p and #2n, where p and n represent the positive and negative masks, respectively. The peaks are indicated by open circles. The time delay difference between mask #2p and #2n is measured as 66 fs in horizontal direction (upper panel) and 0 fs in vertical direction (lower panel), where the acquisition time step was 33 fs. **c**, Masks #1p, #2p and #2n. The reference masks are shown in upper panel and the corresponding images acquired at the position of STEA by CCD are shown in lower panel. **d**, The measured peak time delays in each row of $\boldsymbol{E}(\xi,t)$ as a function of spatial coordinate x (horizontal) and y (vertical). The red solid lines in the upper and the lower panel were linear fits to the relevant experimental data (indicated by blue symbols), which are regarded as the wave fronts in view of y axis and x axis, respectively.

Impact of wave-front tilt on THz ghost imaging

In the premise of guaranteeing the conjugate positions of two DMDs, the optimal correction of wave front is limited by the micromirror tilt angle tolerance of the pair of chosen DMDs, which plays a fatal role in our NGI system.

DMD consists of millions micromirrors and each micromirror is switchable between two discrete positions: -α + β and α + β, where α (12°) represents micromirror tilt angle and β (|β| ≤ ~1°) represents the tilt angle tolerance[1]. Both α and β are measured relative to the plane formed by the overall micromirror array (0° flat reference when the micromirrors are parked in their inactive state), as illustrated in Fig. S6a. By comparing the zero-order diffraction angle in the case of normal incidence, the tilt angle differences between every two DMDs among all three DMDs we have were calculated and are shown in the histogram

in Fig. S6b, which are in agreement with the results deduced from spatiotemporal THz waveform map (symbols with error bar in Fig. S6b) according to the following equation:

$$\left|\theta_{\mathrm{DMD}_i} - \theta_{\mathrm{DMD}_j}\right| = \mathrm{atan}(2\Delta t c / l_{\mathrm{DMD}}), \tag{3.1}$$

where $2\Delta t$ represents the time shift acquired by linear fit to peak time delays, c represents the light speed in air and l_{DMD} represents the DMD coding size in the measuring direction (5535 μm in our cases).

For a visual comparison, the experimental results whose wave front was corrected by DMD_3 and DMD_2 are shown in Figs. S6c-S6e. In this case, the time shift in horizontal was calculated as 385 fs × 2 (corresponding deduced tilt angle difference was 2.39° , which is in agreement with the measurement of 2.45° by trigonometry, see Fig. S6b). In horizontal direction (x), the THz transmission decays too fast from the center to the ends to resolve the spatial information of the object, compared to THz transmission in vertical direction (y), as shown in Fig. S6e. It is worth noting that although the time shift can be completely offset by adjusting the position of DMD slightly away from the conjugate position, the mask patterns cannot be projected clearly simultaneously.

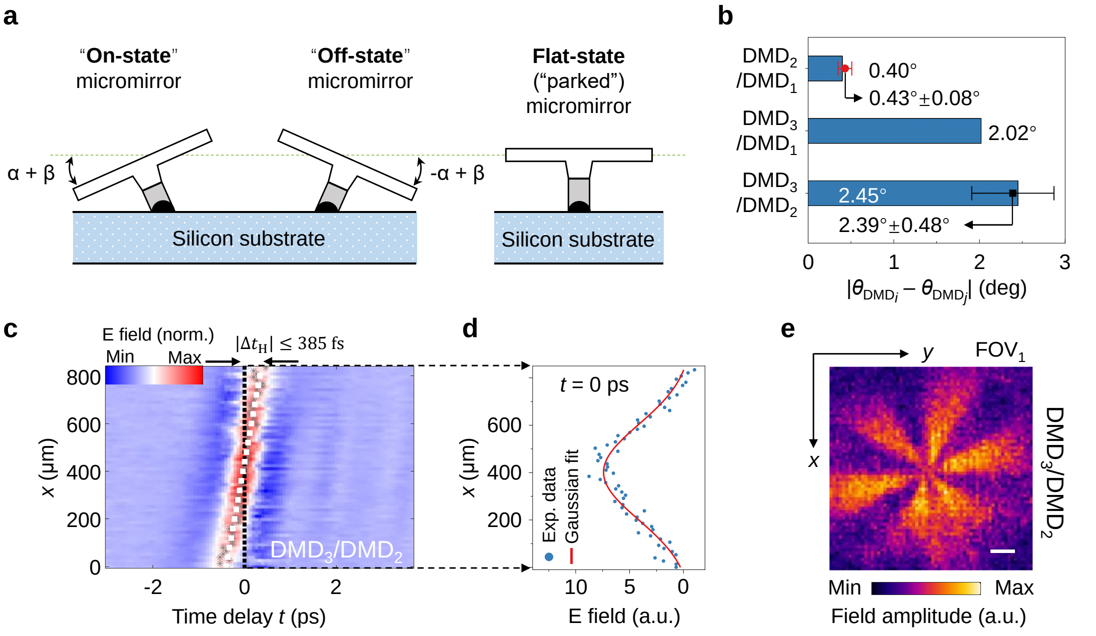

Figure S6| Impact of wave-front tilt angle on THz ghost imaging. a, Micromirror landed positions. Micromirror is landed either -α + β ("off state") or α + β ("on state") in working mode, where α (12°) represents designed micromirror tilt angle and β (|β| ≤ 1°) represents the tilt angle tolerance. Micromirrors would be parked in their inactive state. **b**, Measured tilt angle differences between DMD_i and DMD_j, where subscripts i and j represent 1, 2, 3 and $i \neq j$. The histogram values were measured by trigonometry and the symbols with error bar were deduced from the spatiotemporal THz waveform map. **c**, Experimental spatiotemporal THz waveform map using DMD_2 and DMD_3 to correct wave front tilt. The grey asterisks indicate the peak time delays in every row and the white dotted line is a linear fit to these peak time delays. **d**, Electrical field as a function of horizontal coordinate x at time delay of t = 0 ps (indicated by a black dotted line in **c**). **e**, Corresponding THz near-field ghost image of a cartwheel (fabricated with Au) with FOV_1 = 834 × 834 μm, which was imaged at fixed time

delay of t = 0 ps.

Section 4. Estimation on SNR and potential imaging time of ghost image

Estimation on SNR of ghost image

In practical ghost measurements, the signal vector $\mathbf{Y}$ consisting of N elements can be described as

$$\mathbf{Y} = \mathbf{\Phi O} + \boldsymbol{e}, \tag{4.1}$$

where $\mathbf{O}$ represents the N-length object vector, $\mathbf{\Phi}$ represents an $N \times N$ measurement matrix and $\boldsymbol{e}$ represents an N-length error vector whose each element is associated with each measurement.

The ghost image can be reconstructed by

$$\mathbf{X} = \mathbf{\Phi}^{-1}\mathbf{Y} = \mathbf{\Phi}^{-1}(\mathbf{\Phi O} + \boldsymbol{e}) = \mathbf{O} + \mathbf{\Phi}^{-1}\boldsymbol{e}. \tag{4.2}$$

The root mean square error σ across all pixels can be calculated by

$$\sigma = \mathrm{STD}[\mathbf{X} - \mathbf{O}] = \mathrm{STD}[\mathbf{\Phi}^{-1}\boldsymbol{e}], \tag{4.3}$$

where $\mathrm{STD}[\mathbf{\Phi}^{-1}\mathbf{e}]$ represents the standard deviation of the vector in the square brackets. We assume that average value of all elements in $\boldsymbol{e}$ is 0 with a standard deviation of σ_e, and that the errors in individual measurements are independent. Then the Equation (4.3) can be written as

$$\sigma = \sigma_e\sqrt{\frac{\mathrm{TRA}[(\mathbf{\Phi}^{\mathrm{T}}\mathbf{\Phi})^{-1}]}{N}}, \tag{4.4}$$

Where $\mathrm{TRA}[(\mathbf{\Phi}^{\mathrm{T}}\mathbf{\Phi})^{-1}]$ represents the trace of the square matrix in the square brackets, and the superscript T represents the transpose of its corresponding matrix. Then the signal-to-noise ratio of the image can be calculated by

$$\mathrm{SNR} = \frac{\mu}{\sigma} = \frac{E_0}{N\sigma_e}\sqrt{\frac{N}{\mathrm{TRA}[(\mathbf{\Phi}^{\mathrm{T}}\mathbf{\Phi})^{-1}]}}, \tag{4.5}$$

where E_0 represents the total energy for imaging. It has been demonstrated that $\mathrm{TRA}[(\mathbf{\Phi}^{\mathrm{T}}\mathbf{\Phi})^{-1}] \geq 1$ with the equality being reached if and only if $\mathbf{\Phi}$ is a Hadamard matrix[2]. In this case, Equation (4.5) can be written as

$$\mathrm{SNR_H} = \frac{E_0}{\sigma_e\sqrt{N}}. \tag{4.6}$$

But in the case of raster scanning, the measurement matrix $\mathbf{\Phi}$ is an identity matrix of order N. Then the Equation (4.5) can be written as

$$\mathrm{SNR_R} = \frac{E_0}{\sigma_e N}. \tag{4.7}$$

It is worth noting that σ_e is dependent on the multiplexing method $\mathbf{\Phi}$. In our pulsed imaging system, the noises consist of dark noises σ_d from detector and source fluctuation σ_s among pulses. Here we define two dimensionless parameters $\gamma_d \equiv \sigma_d/E_0$ and $\gamma_s \equiv \sigma_s/E_0$ to describe the dark-noise-to-peak ratio and source-fluctuation-to-peak ratio, respectively. In the case of Hadamard multiplexing, half of the incident light is used for each measurement (apart from the pair of first positive and negative measurement), and then $\sigma_{e,H}$ can be calculated by

$$\sigma_{e,H} = \sqrt{\frac{2}{k}}\sqrt{\sigma_d^2 + \left(\frac{\sigma_s}{2}\right)^2} = E_0\sqrt{\frac{2}{k}\left[\gamma_d^2 + (\gamma_s/2)^2\right]}, \tag{4.8}$$

where k represents the number of measurements for each mask and the product term $\sqrt{2}$

indicates that each measurement value in vector $\mathbf{Y}$ is a subtraction of one negative measurement from its corresponding positive measurement. While in the case of raster scanning, only E_0/N of the incident light is used for each measurement (N is usually greater than 1000), so the source fluctuation is negligible compared to the dark noise, and then $\sigma_{\mathrm{e,R}}$ can be calculated by

$$\sigma_{\mathrm{e,R}} = E_0\gamma_{\mathrm{d}}\sqrt{\frac{1}{2k}}, \tag{4.9}$$

where the number of measurements for each scanning mask is $2k$, for fair comparison in the term of acquisition time. With Equations (4.6) - (4.9), the $\mathrm{SNR_H}$ and the $\mathrm{SNR_R}$ can be calculated by

$$\mathrm{SNR_H} = \sqrt{\frac{k}{2[\gamma_{\mathrm{d}}^2+(\gamma_s/2)^2]N}}, \tag{4.10}$$

$$\mathrm{SNR_R} = \frac{\sqrt{2k}}{\gamma_{\mathrm{d}}N}. \tag{4.11}$$

Here, we simulated the noised ghost images multiplexed using Hadamard matrix with experimental parameters ($\gamma_{\mathrm{d}} = 10^{-3}$, $\gamma_{\mathrm{s}} = 7 \times 10^{-3}$, and $k = 15$), which are shown in Fig. S7. The raster scanning results are also given for comparison. The SNRs calculated from the reconstructed images meet agreement with the corresponding SNRs estimated via Equations (4.10) and (4.11), which are shown at the bottom of Fig. S7b-S7g.

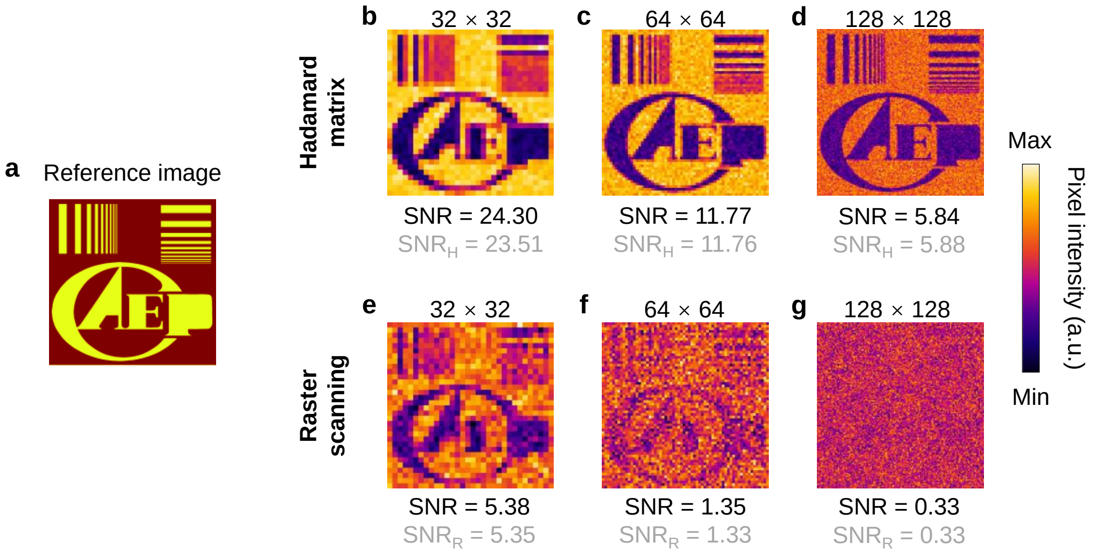

Figure S7| Simulated ghost images. a, The reference image. **b-d**, Simulated noised ghost images multiplexed using Hadamard matrix with order of 32 × 32 (**b**), 64 × 64 (**c**), and 128 ×128 (**d**). The results were simulated with parameters of $\gamma_{\mathrm{d}} = 10^{-3}$, $\gamma_s = 7 \times 10^{-3}$, and k = 15. **e-g**, Simulated noised ghost images multiplexed using identity matrix (raster-scanning matrix) with order of 32 × 32 (**e**), 64 × 64 (**f**), and 128 ×128 (**g**). The results were simulated with the same parameters as in the case of Hadamard multiplexing. The SNRs calculated from the reconstructed images are given at the bottom of each image, and the estimated SNRs are also given (grey) for comparison.

Estimation on the potential frame rate of ghost image

The imaging speed of a GHOSTEAM driven by a MHz oscillator can be enhanced

significantly, due to three facts:

1) MHz oscillator can provide minimal acquisition time for single mask (t_{mask}). The shorter t_{mask} is, the faster frame rate (FPS) can be achieved. The minimal t_{mask} is limited by the pulse period t_0. The pulse period of oscillator is ~10 ns (e.g., 12.5 ns for an 80-MHz oscillator, see Fig. S8), which is five orders of magnitude smaller than the kHz-amplifier pulse period. But it is worth noting that besides pulse period t_0, minimal t_{mask} is limited by the DMD switching time as well, which has typical values of 44-90 μs (depends on the specific DMD type). So, the MHz oscillator can provide minimal t_{mask} at the scale of several hundreds of microseconds.
2) MHz oscillator can provide much more stable illumination for ghost imaging within the identical acquisition time, compared to kHz amplifier. A stable illuminating source can provide ghost images with the same quality (SNR) in much shorter acquisition time. The fluctuation ratio among pulses ("integration time" of 12.5 ns for each data) from oscillator was measured as $\gamma_{s0} = 3.8 \times 10^{-3}$, as shown in Fig. S8.
3) MHz oscillator can efficiently excite the STE to radiate THz pulses with a demonstrated dynamic range of more than 60 dB (1000:1)[3]. The intense illuminating source can also provide ghost images with the same quality (SNR) in much shorter acquisition time.

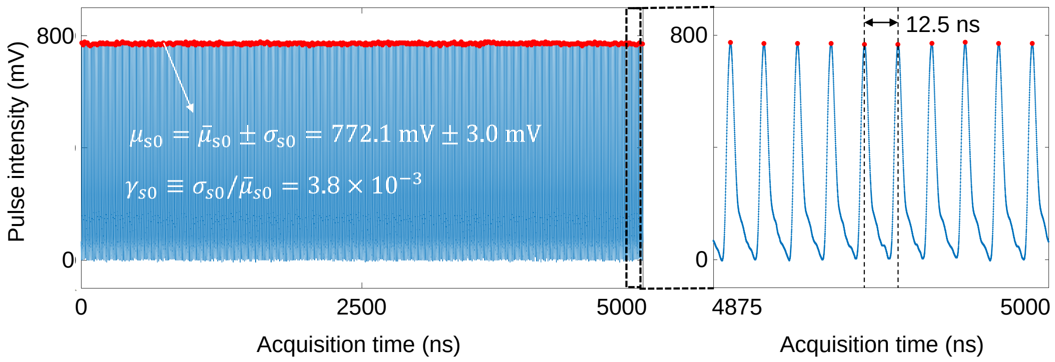

Figure S8| Laser pulses from 80 MHz oscillator. The fluctuation ratio among pulses γ_{s0} was measured as 3.8×10^{-3} from the recorded 400 pulses.

The acquisition time for one ghost image via N-order Hadamard multiplexing equals $2Nt_{mask}r_c$, where r_c represents the compressive ratio. The frame rate then can be calculated by:

$$\mathrm{FPS} = (2Nt_{\mathrm{mask}}r_{\mathrm{c}})^{-1}. \tag{4.12}$$

Within the "integration time" of t_{mask}, there are

$$\gamma_{\mathrm{d}}(t_{\mathrm{mask}}) = \gamma_{\mathrm{d0}}\sqrt{t_0/t_{\mathrm{mask}}}, \tag{4.13}$$

$$\gamma_{\mathrm{s}}(t_{\mathrm{mask}}) = \gamma_{\mathrm{s0}}\sqrt{t_0/t_{\mathrm{mask}}}, \tag{4.14}$$

Where γ_{d0} and γ_{s0} denote the ratio of dark noise to single THz peak and pulse fluctuation ratio within the "integration time" of pulse period t_0, respectively. In combination with Equation (4.10), the signal-to-noise ratio of ghost image via Hadamard multiplexing is:

$$\mathrm{SNR_H} = \sqrt{\frac{t_{\mathrm{mask}}}{2N(\gamma_{d0}^2+\gamma_{s0}^2/4)t_0}}. \tag{4.15}$$

The acquisition time for single mask can be expressed as a function of $\mathrm{SNR_H}$ and N:

$$t_{\mathrm{mask}} = 2N(\gamma_{d0}^2 + \gamma_{s0}^2/4)t_0\mathrm{SNR_H^2}. \tag{4.16}$$

The imaging time can be calculated as:

$t_{\mathrm{GI}} = 2Nt_{\mathrm{mask}}$.(4.17)In the GHOSTEAM driven by a typical 80-MHz oscillator, the pulse period t_0 equals 12.5 ns, and the pulse fluctuation ratio γ_{s0} was measured as 3.8×10^{-3}, as shown in Fig. S8.

In Ref. [3], the researchers use a 75-MHz oscillator to drive a spintronic THz emitter (pump energy of 4.7 nJ, pump spot diameter of ~10 μm) and obtained THz signals whose spectral dynamic range is well above 60 dB (1000:1) with a maximum around 1.5 THz. The THz signals were detected by a dipole GaAs photoconductive antenna with integration time of 100 ms and the corresponding spectra were not corrected for the detector response. Thus, the dark-noise-to-peak ratio of $(1000)^{-1}$ can be reached with typical integration time of 100 ms, namely $\gamma_{\mathrm{d}}(100\ \mathrm{ms}) = 1 \times 10^{-3}$.

With these parameters and $\mathrm{SNR_H}$ = 11.76, the estimated imaging time with $N = 16 \times 16$, 32 × 32 and 64 × 64 are 3.6 s, 58 s, and 928 s (~15 min), respectively, as shown in Fig. S9.

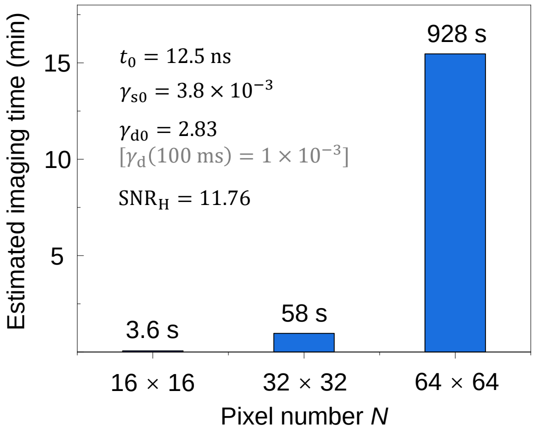

Figure S9| Estimated imaging time with different *N*. The ghost imaging time was calculated using Equation (4.17) with parameters given in the figure.

Section 5. Analysis of the experimental spatial resolution

Boltzmann sigmoidal function

The Boltzmann sigmoidal function subjected to

$$E(x) = \frac{E_1 - E_2}{1 + e^{(x - x_0)/dx}} + E_2, \tag{5.1}$$

is used to fit the experimental data in Fig. 2d.

Three sample curves with different $\mathrm{d}x$ ($E_1 = 0$, $E_2 = 1$ and $x_0 = 0$) are given in Fig. S10a. The edge sharpens with reducing $\mathrm{d}x$. According to a 10%-90% criterion of maximum electrical field amplitude[4], the spatial resolution can be quantified.

Fit to experimental data

In Fig. 2e of the main text, the *y*-dependent field amplitudes $E(y)$ were individually averaged from the pixels in each row $[E(x_a,y), E(x_a+\Delta x,y), \ldots, E(x_b,y)]$, where x_a and x_b represent the starting and end pixel position relevant to the slit region, respectively. And the solid curves are reconstructed using piecewise Boltzmann sigmoidal functions with parameters $E_1 = 1.90\ (0.16)$, $E_2 = 0.16\ (1.90)$, $\mathrm{d}x$ = 0.2276 μm and x_0 being a vector consisted of the positions decided by the metal edges. These parameters were derived

from the experimental fits to the first four edges, as described below.

We have fitted the first four edges (Fig. S10b) and acquired four high-level amplitude values with average of 1.90 and four low-level amplitude values with average of 0.16. Meanwhile the $x_0^{(1)} = 74.97$ μm, $x_0^{(2)} = 112.23$ μm, $x_0^{(3)} = 153.62$ μm, $x_0^{(4)} = 182.83$ μm were acquired, where the subscript indicates the number of the fitted curve. Associated with metal geometry (the slit widths indicated in Fig. 2e), $x_0^{(1)} = 73.42 \pm 1.18$ μm was determined.

According to a 10%—90% criterion of maximum electrical field amplitude, the edge length is calculated as $\sim$1 μm when $\mathrm{d}x$ equals 0.2276 μm.

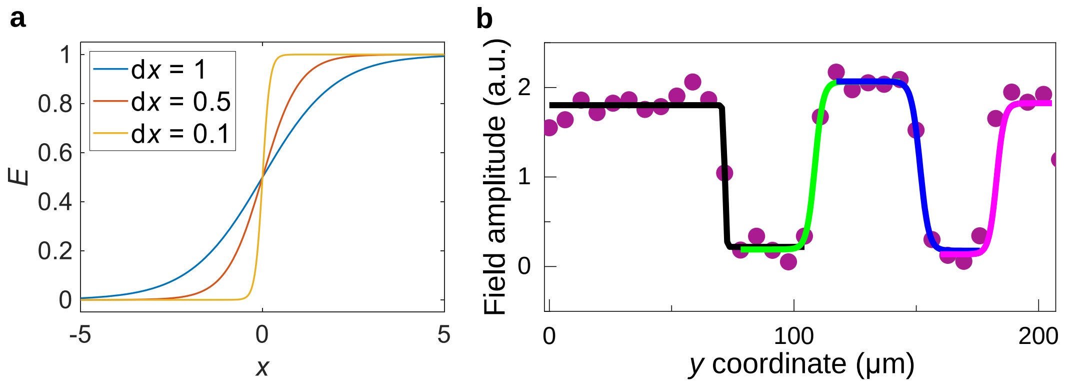

Figure S10| Analysis of the experimental spatial resolution. a, Boltzmann sigmoidal sample curves with different $\mathrm{d}x$. **b**, Piecewise Boltzmann sigmoidal fits to the first four metal edges.

Calculation of the contrast

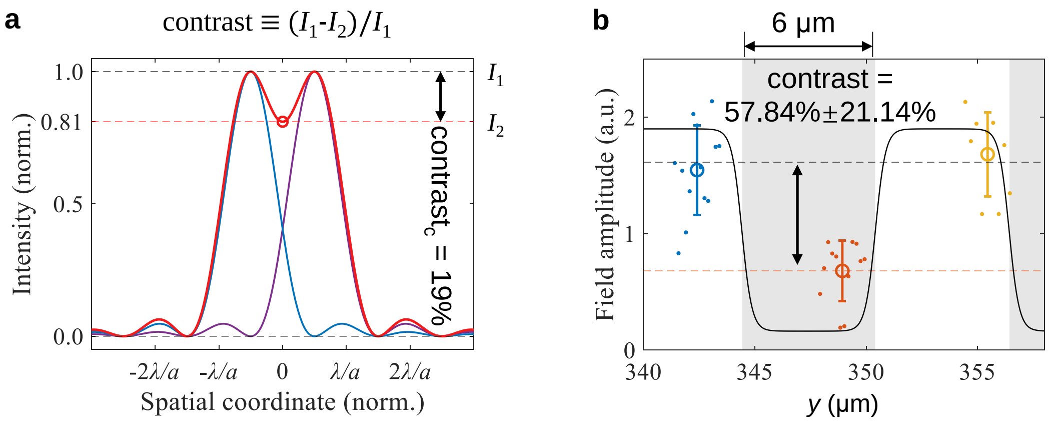

Figure S11| Contrast calculation. a, Intensity distribution of the light diffracted from two slits with normalized distance of 0.61λ/a, where λ denotes the wavelength and a denotes the slit width. The critical contrast of a slit equals 19%, according to the Rayleigh criterion. **b**, The calculated contrast of 57.84% $\pm$ 21.14% at the narrowest 6-μm metal slit. The field amplitudes of the markers from left to right are 1.55 $\pm$ 0.38 (blue), 0.74 $\pm$ 0.32 (orange) and 1.68 $\pm$ 0.36 (yellow), respectively. The black curves are plotted using the reconstructed piecewise Boltzmann sigmoidal functions (as described above).

Section 6. Data acquisition and processing for THz near-field ghost imaging

Walsh-Hadamard matrix $\mathbf{\Phi}$ consisting of -1 and 1 was realized by equation

$$\mathbf{\Phi} = \mathbf{\Phi}_{\mathrm{p}} - \mathbf{\Phi}_{\mathrm{n}}. \tag{6.1}$$

To complete a 64 × 64 ghost image, 4096-order Walsh-Hadamard matrix was used to code the masks that displayed on DMD_1. The signal vector $\mathbf{Y}$ with length of 4096 was measured as

$$\mathbf{Y} = \mathbf{Y}_{\mathrm{p}} - \mathbf{Y}_{\mathrm{n}}, \tag{6.2}$$

where $\mathbf{Y}_{\mathrm{p}}$ and $\mathbf{Y}_{\mathrm{n}}$ represent a 4096-lengthed vector consisting of positive mask values and negative mask values, respectively, as illustrated in Fig. S12a. The ghost image can be eventually reconstructed by

$$\mathbf{X} = \mathbf{\Phi}^{-1}\mathbf{Y}. \tag{6.3}$$

To minimize the low-frequency amplitude oscillations arise from slow shift of laser power, the negative masks were performed immediately after corresponding positive masks (Fig. S12b). To suppress the high-frequency amplitude oscillations arise from detector noises and fluctuation among laser pulses, each mask value was averaged by 15 valid data (grey symbols but the ones in rising edge and falling edge, in Fig. S12b). These data were recorded by a lock-in amplifier with integration time of 100 ms. The energy of $\mathbf{Y}$ is mainly centralized to mask patterns with low spatial frequencies (e.g., the neighboring masks of mask #1, #128, #256, ···, as shown in Fig. S12c).

One can see Supplementary GIFs. 3-5 for the ghost images reconstructed with sub-sample data.

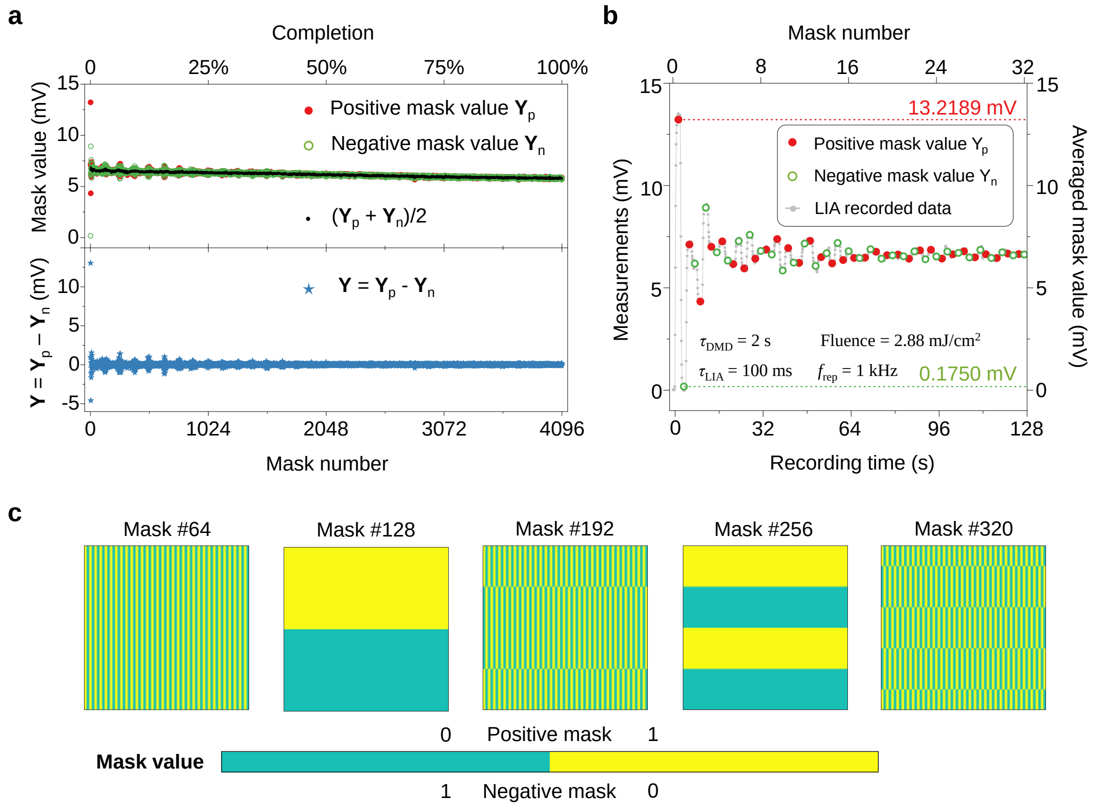

Figure S12| Data acquisition and processing for THz Near-field ghost imaging. a, The full measurements for Fig. 2b. **b**, The first 32 mask values (positive and negative) recorded by lock-in amplifier (LIA). Negative mask measurements were performed immediately after

corresponding positive mask measurements, which can minimize low-frequency source noise. Pulse fluence (repetition rate of 1 kHz) on spintronic THz emitter was 2.88 mJ cm^{-2}. Each mask was displayed on DMD_1 for τ_{DMD} = 2 s and each datum (transmitted THz field) was recorded with LIA integration time of τ_{LIA} = 100 ms. **c**, Reference masks of #64, #128, #192, #256 and #320 coded by 4096-order Walsh-Hadamard matrix.

Section 7. Details of image fusion

The fused images are assessed in terms of clearness and directional spatial information amount, respectively.

Clearness of image

Signal-to-noise ratio (SNR) is a widely used assessment parameter for image's quality. The object is known to be binary, and so we choose two regions, one representing a metal area and the other representing a transparent area to calculate the SNR

$$\mathrm{SNR} = \mu/\sigma, \tag{7.1}$$

where μ is the mean pixel intensity in the transparent region and σ is the standard deviation of the pixel intensity in the metal region. In our experiment, the regions were defined as indicated in Fig. S13a, and corresponding SNR of *x*-polarized (Fig. 2b), *y*-polarized (Fig. 2c) and fused (Fig. 3d) images are shown in Fig. S13b. The calculated SNR meets an agreement with the estimated value within experimental parameters, as illustrated in Fig. 1f.

Total variation (TV) is another common assessment parameter for image's clearness. Compared to calculation of SNR, calculation of TV does not need priori knowledge of the object, because TV is calculated from global information according to

$$\mathrm{TV} \equiv \left(\sum_{i=2}^{L_1}\sum_{j=2}^{L_2}[(\nabla_{\mathrm{H}}\mathbf{X}(i,j))^2 + (\nabla_{\mathrm{V}}\mathbf{X}(i,j))^2]\right)^{1/2}, \tag{7.2}$$

where $\mathbf{X}$ represents the image with $L_1 \times L_2$ pixels, ∇_{H} and ∇_{V} are the discretized gradient operators along the horizontal and vertical directions, respectively.

Amount of image's spatial information

The pixels' intensity variation in spatial domain represents the amount of image's spatial information. Due to fact that the THz images were acquired with either $\boldsymbol{E}_{\mathrm{THz}} = |\boldsymbol{E}_0|\hat{\boldsymbol{e}}_x$ or $\boldsymbol{E}_{\mathrm{THz}} = |\boldsymbol{E}_0|\hat{\boldsymbol{e}}_y$, there should be a significant difference between horizontal and vertical spatial information amount. Two spatial gradients with mutually orthogonal directions[5] are used to quantify the image's spatial information

$$G_{\mathrm{row}} \equiv \left(\sum_{i}^{L_1}\sum_{j=2}^{L_2}[(\nabla_{\mathrm{H}}\mathbf{X}(i,j))^2]\right)^{1/2}, \tag{7.3}$$

$$G_{\mathrm{col}} \equiv \left(\sum_{i=2}^{L_1}\sum_{j}^{L_2}[(\nabla_{\mathrm{V}}\mathbf{X}(i,j))^2]\right)^{1/2}. \tag{7.4}$$

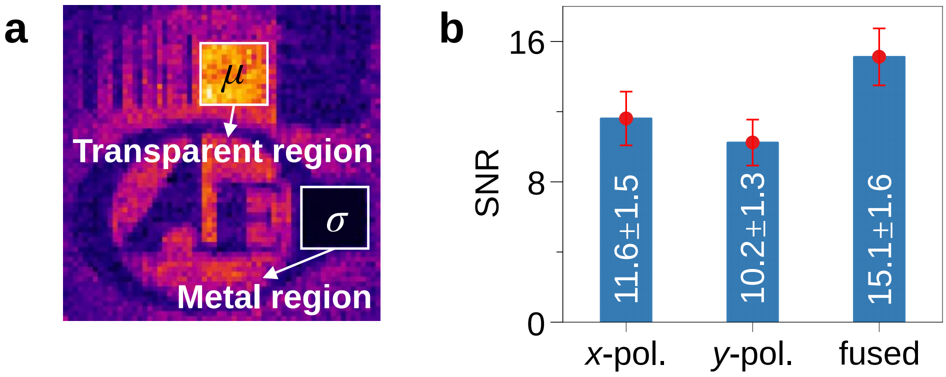

Figure S13| Calculation of images' SNR. a, Transparent and metal regions defined for experimental SNR calculation. **b**, Calculated SNRs of x-polarized, y-polarized and fused (weight fraction of $r = 0.53$) images.

References

1. T.I. DLP4500 0.45WXGA DMD. T.I. [Specification] 2016 [cited DLPS 028B]; 2013-Apr:[57]. Available from: http://www.ti.com/lit/ds/symlink/dlp4500.pdf

2. Harwit M, Sloane NJA. Chapter 3 – The Basic Theory of Hadamard Transform Spectrometers and Imagers. *Hadamard Transform Optics*. Academic Press, 1979, pp 44-95.

3. Torosyan G, Keller S, Scheuer L, Beigang R, Papaioannou ET. Optimized Spintronic Terahertz Emitters Based on Epitaxial Grown Fe/Pt Layer Structures. *Sci Rep* 2018, **8**(1)**:** 1311.

4. Kawano Y, Ishibashi K. An on-chip near-field terahertz probe and detector. *Nature Photonics* 2008, **2**(10)**:** 618-621.

5. Eskicioglu AM, Fisher PS. Image quality measures and their performance. *IEEE Transactions on Communications* 1995, **43**(12)**:** 2959-2965.
