# Supplementary figures and images for: Ghost spintronic THz-emitter-array microscope
Source: Light Sci Appl. 2020 Jun 8;9:99. doi: 10.1038/s41377-020-0338-4 (PMC7280226; doi:10.1038/s41377-020-0338-4)

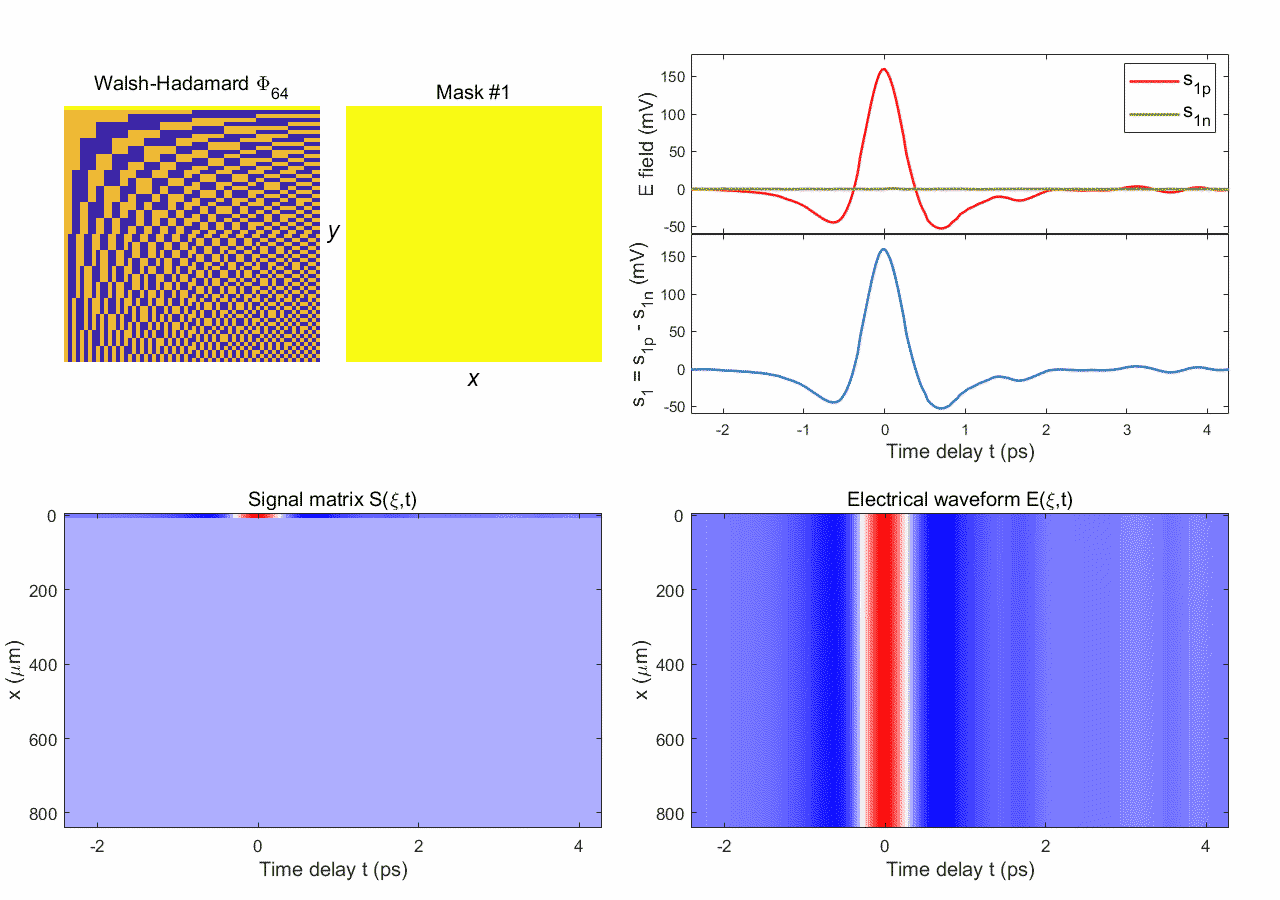

Supplement: Supplementary file 2 — Supplementary-GIF1 [file 41377_2020_338_MOESM2_ESM.gif]

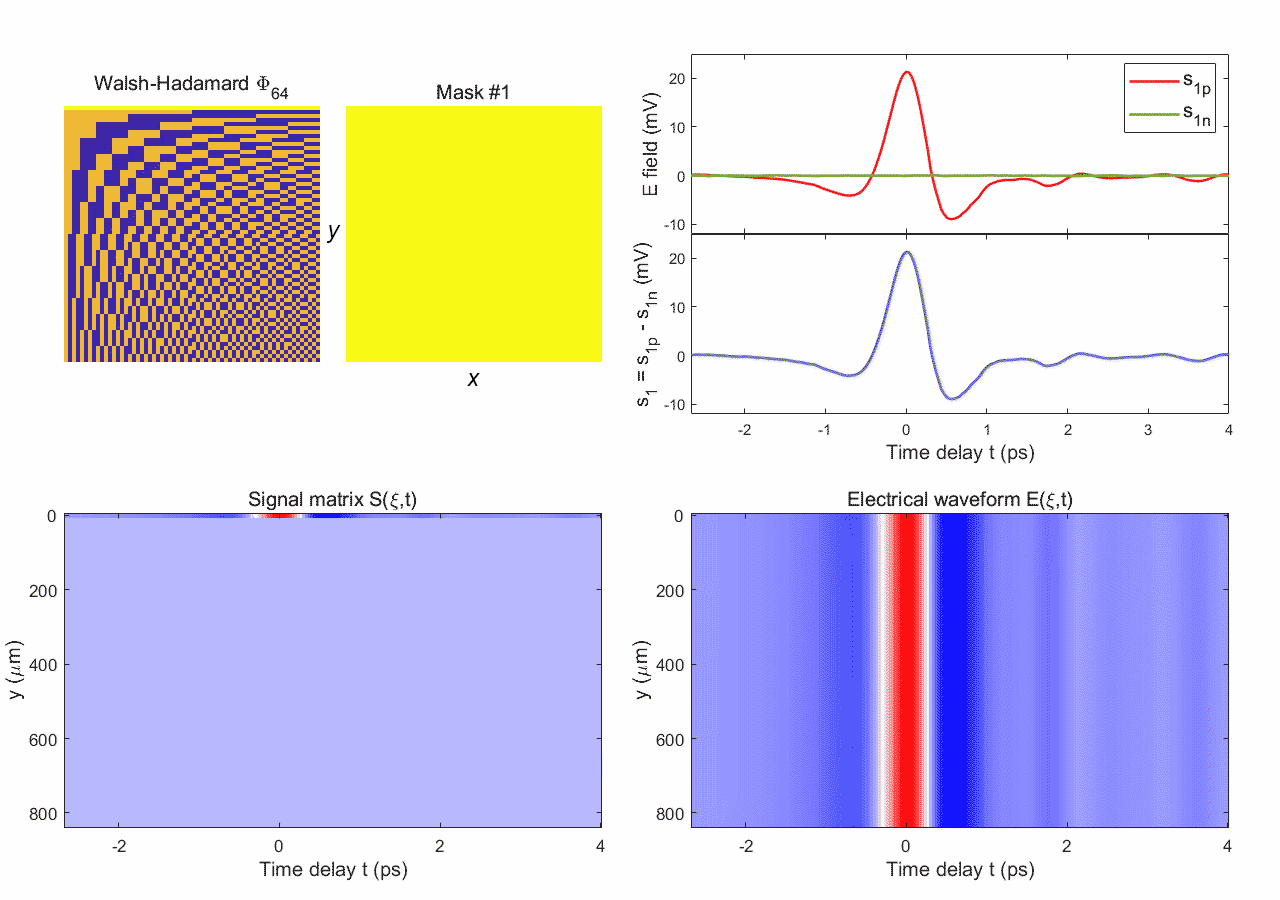

Supplement: Supplementary file 3 — Supplementary-GIF2 [file 41377_2020_338_MOESM3_ESM.gif]

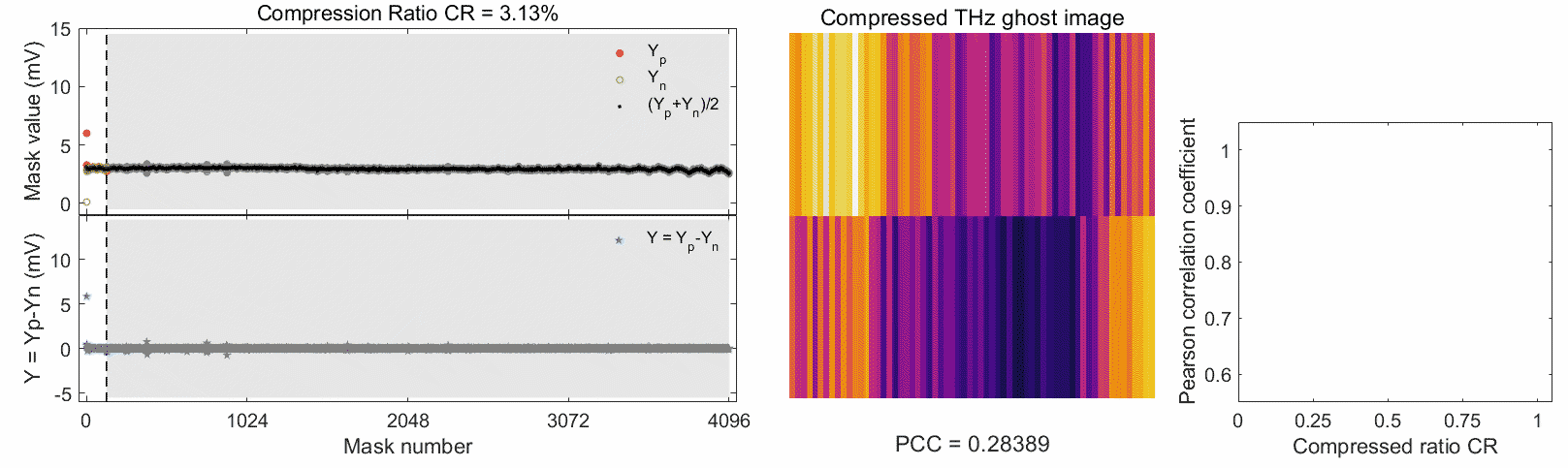

Supplement: Supplementary file 4 — Supplementary-GIF3 [file 41377_2020_338_MOESM4_ESM.gif]

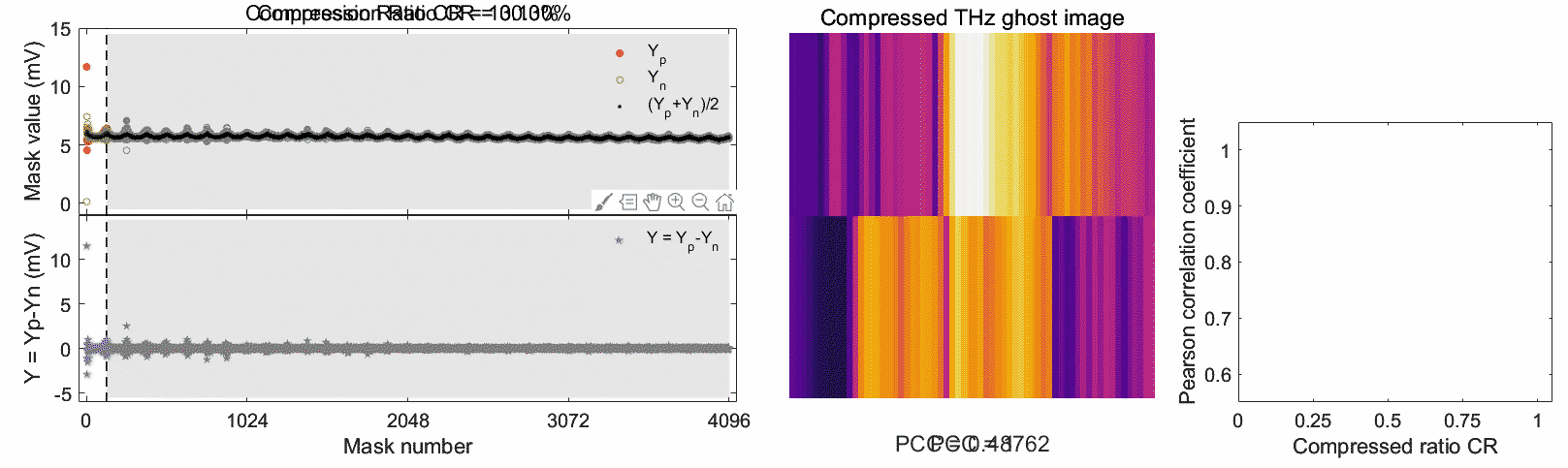

Supplement: Supplementary file 5 — Supplementary-GIF4 [file 41377_2020_338_MOESM5_ESM.gif]

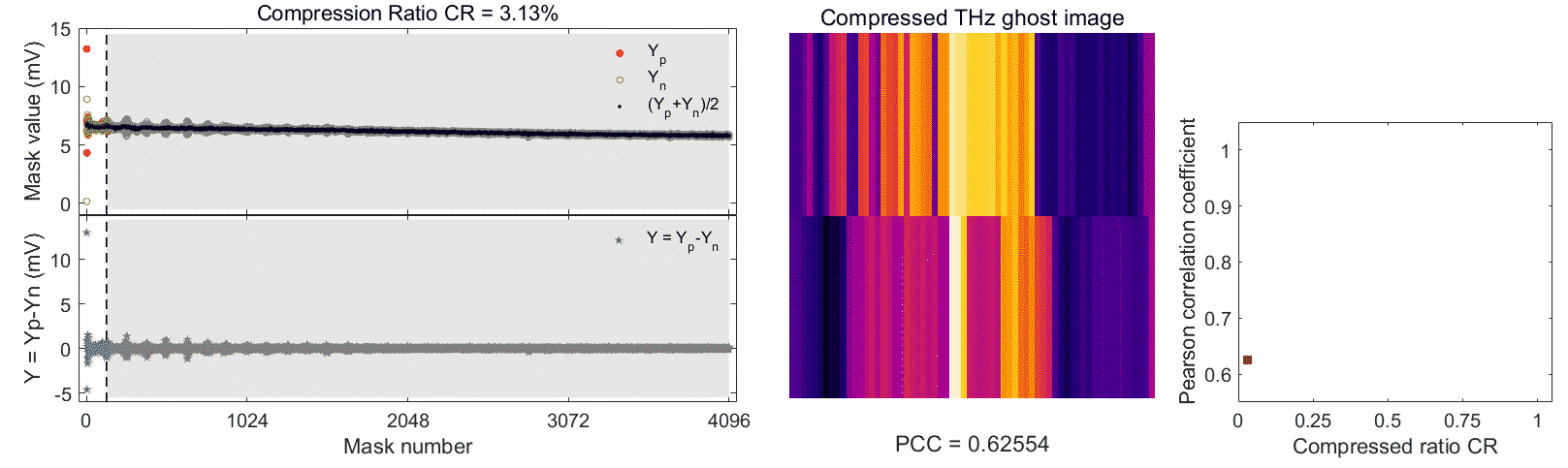

Supplement: Supplementary file 6 — Supplementary-GIF5 [file 41377_2020_338_MOESM6_ESM.gif]
